# Current state of research on the clinical benefits of herbal medicines for non-life-threatening ailments

**DOI:** 10.3389/fphar.2023.1234701

**Published:** 2023-09-28

**Authors:** Sandra Salm, Jochen Rutz, Marjan van den Akker, Roman A. Blaheta, Beatrice E. Bachmeier

**Affiliations:** ^1^ Institute of Pharmaceutical Biology, Goethe University, Frankfurt, Germany; ^2^ Institute of General Practice, Goethe University, Frankfurt, Germany; ^3^ Department of Urology and Pediatric Urology, University Medical Center Mainz, Mainz, Germany; ^4^ Department of Family Medicine, Care and Public Health Research Institute, Maastricht University, Maastricht, Netherlands; ^5^ Department of Public Health and Primary Care, Academic Centre of General Practice, KU Leuven, Leuven, Belgium

**Keywords:** herbal medicine, clinical benefits, psychosomatic disorders, gynecological complaints, gastrointestinal disorders, urinary tract infections, upper respiratory tract infections

## Abstract

Herbal medicines are becoming increasingly popular among patients because they are well tolerated and do not exert severe side effects. Nevertheless, they receive little consideration in therapeutic settings. The present article reviews the current state of research on the clinical benefits of herbal medicines on five indication groups, psychosomatic disorders, gynecological complaints, gastrointestinal disorders, urinary and upper respiratory tract infections. The study search was based on the database PubMed and concentrated on herbal medicines legally approved in Europe. After applying defined inclusion and exclusion criteria, 141 articles were selected: 59 for psychosomatic disorders (100% randomized controlled trials; RCTs), 20 for gynecological complaints (56% RCTs), 19 for gastrointestinal disorders (68% RCTs), 16 for urinary tract infections (UTI, 63% RCTs) and 24 for upper respiratory tract infections (URTI) (79% RCTs). For the majority of the studies, therapeutic benefits were evaluated by patient reported outcome measures (PROs). For psychosomatic disorders, gynecological complaints and URTI more than 80% of the study outcomes were positive, whereas the clinical benefit of herbal medicines for the treatment of UTI and gastrointestinal disorders was lower with 55%. The critical appraisal of the articles shows that there is a lack of high-quality studies and, with regard to gastrointestinal disorders, the clinical benefits of herbal medicines as a stand-alone form of therapy are unclear. According to the current state of knowledge, scientific evidence has still to be improved to allow integration of herbal medicines into guidelines and standard treatment regimens for the indications reviewed here. In addition to clinical data, real world data and outcome measures can add significant value to pave the way for herbal medicines into future therapeutic applications.

## 1 Introduction

Plant derived drugs have been used since humans have started treating physical and mental illnesses. They are part of Traditional Medicine in different cultures all over the world (Yuan et al., 2016). Since then, medicine and treatment procedures have evolved and while in Traditional Medicine a holistic approach of life focusing on health and its maintenance was common philosophy, present Modern Medicine has a clear emphasis on unravelling the changes leading to disease and eradiating it (Fries, 2019). Traditional medicine has a rigorous algorithm of identifying the root of the disease, which is based on traditional concepts, which, unfortunately, are considered obsolete nowadays, despite their practical longevity (e.g., acupuncture, ayurveda). The problem is that this traditional medical epistemology is not fully understood and science has limited tools to “translate” it into modern terms.

With the success of synthetic drugs along with the design of targeted therapies interfering specifically with the respective disease-related signaling pathways, herbal medicines have been eliminated from modern rational treatment strategies. The most important obstacles for the use in novel therapy strategies is that markers to measure clinical efficacy of herbal medicine have not been developed so far. Markers of efficacy of herbal drugs could also be useful to distinguish between patients who could benefit from a therapy with herbal medicines from those who will not. First preclinical studies already indicate that those markers or “signatures” (e.g., mRNA, miRNA) could be found in the future (Bachmeier et al., 2007; Bachmeier et al., 2008; Bachmeier et al., 2009; Bachmeier et al., 2010; Killian et al., 2012; Kronski et al., 2014).

In the last years, more and more patients report on the perceived efficacy of herbal drugs and praise the absence of undesired side effects and the good tolerability.

The following section provides insights into the standard therapies of selected ailments for which herbal medicines may be a rational alternative.

### 1.1 Indications suitable for treatment with herbal medicines

Herbal medicines are in particular suitable for the treatment of non-life-threatening conditions for which knowledge from traditional use is available pointing to their clinical benefits in treating the respective ailment (Wachtel-Galor and Benzie, 2011). This applies especially to psychosomatic disorders, gynecological complaints, and upper respiratory tract infections. However also for other diseases like gastrointestinal diseases, urinary tract infections herbal medicines have been clinically applied and—as we will show in this review—with some success.

Standard Care of psychosomatic disorders comprises the application of synthetic psychotropic drugs and psychotherapy (Laux, 2021). Psychotropic drugs are used not only for the treatment of depressive disorders and anxiety, but also for sleep disorders, excitation and chronic pain (Gründer and Benkert, 2012). However undesired adverse events having negative impact on quality of life can occur like, e.g., weight gain, sexual dysfunction, sedation, headache and tremor (Grunze et al., 2017). In addition their use, in particular benzodiazepines, can lead to addiction and drug abuse (Soyka and Mann, 2018) and interactions with other medication has to be taken into consideration especially in older multimorbid patients (Burkhardt and Wehling, 2010). About 23% of all over 70-year-old people have psychosomatic disorders with about 40% requiring therapy (Haupt and Vollmar, 2008). In this context herbal medicines represent an interesting alternative to avoid the above-mentioned problems with standard synthetic drugs. However, they do not belong to standard therapy-options and therefore are underrepresented in therapy-guidelines (Bittel et al., 2022). Nevertheless they play an important role in self-medication of patients (Stange, 2014) probably due to their favorable ratio between benefit and side-effects.

Gynecological complaints include, e.g., menopausal and premenstrual symptoms. According to the German medical guideline for post- and perimenopause, vasomotor symptoms of the peri- and post-menopause such as hot flushes and sweating should be treated with hormone therapy for menopause (hormone replacement therapy; HRT), if not contraindicated (AWMF, 2020). The side effects of HRT include edema, joint pain, psychological symptoms or even thrombosis and breast cancer (Maclennan et al., 2004). Herbal medicines, on the other hand, are characterized by a low risk of adverse events which increases patients’ adherence and in consequence prevents therapy discontinuations (AWMF, 2020). Premenstrual syndrome (PMS) is characterized by recurring physical and psychological symptoms in the days before menstruation. There are currently no medical guidelines in German-speaking countries for the treatment of PMS. Systematic reviews on hormonal treatments (oral contraceptives, progesterone and estrogen) (Ford et al., 2006; Lopez et al., 2007; Naheed et al., 2013; Kwan and Onwude, 2015) and acupuncture/acupressure (Armour et al., 2018) point to ambiguous evidence. Treatment with serotonin reuptake inhibitors was shown to be effective but was associated with frequent side effects, e.g., nausea and asthenia (Marjoribanks et al., 2013).

Gastrointestinal diseases include several conditions like irritable bowel syndrome (IBS), inflammatory bowel disease (IBD), liver disease (hepatitis), and functional dyspepsia (FD).

Beside dietary changes, stress management and psychotherapy, severe cases of IBS and IBD require additional medication to reduce inflammation or to slow down the intestinal irritations. However patients often complain about the side effects of medical treatment like, e.g., dizziness or weight gain (particularly caused by steroids), or undesired fatigue, headache, and/or tiredness associated with the intake of methotrexate (Feagan et al., 1995). Common types of hepatitis are viral hepatitis B and C. Antiviral therapy represents the treatment of choice to fight the virus caused disease. However, poor tolerability and significant adverse effects that include, for example, headaches, dizziness, depression, and irritability often lead to treatment discontinuation, further decreasing response rates (Cornberg et al., 2002). FD is a common gastrointestinal disorder treated by proton pump inhibitors (PPI) or H2 receptor antagonist, and/or treatment with tricyclic antidepressants or prokinetic agents. As in all cases, adverse side effects may occur ranging from dizziness to the development of diabetes mellitus type 2 (Yuan et al., 2021).

Urinary tract infections (UTI) with estimated 150 million cases worldwide each year reflect the most common outpatient infections (Zavala-Cerna et al., 2020). Women are more susceptible than men with a lifetime incidence of 50%–60%. Application of antibiotics represents the standard treatment regimen to overcome the infection. However, serious side effects, predominantly exerted on the digestive system, may outweigh the benefits of this drug class. Most importantly, routine use of antibiotics bears the risk to trigger the selection of resistant strains. Hence, avoiding antibiotic treatment of UTI has gained high priority among the urologic community (Jung et al., 2023). Lower urinary tract symptoms (LUTS) caused by benign prostatic hyperplasia (BPH) requires a medical therapy which aims to reduce the BPH-related complications. A range of synthetic drugs is available to treat this condition. However, these have a range of side effects, including postural hypotension, dizziness, asthenia, abnormal ejaculation, intraoperative floppy iris syndrome (α1-blocker), or decreased libido, gynecomastia, and erectile dysfunction (5α-reductase inhibitors) (Cheng et al., 2020). Due to this, patients often discontinue treatment.

The most common acute upper respiratory infections include bronchitis, rhinosinusitis and common cold. Common cold or acute viral rhinosinusitis is triggered by a viral infection/inflammation of the nose and by definition has a duration up to 10 days. According to Jaume and co-workers (Jaume et al., 2020) the recommended therapy (mainly symptomatic) contains of paracetamol, NSAIDs, second-generation antihistamines to reduce symptoms the first 2 days; nasal decongestants with small effect in nasal congestion in adults; combination of analgesics and nasal decongestants; ipratropium bromide for reducing rhinorrhea; probiotics; zinc when administered the first 24 h after the onset of symptoms; nasal saline irrigations; and some herbal medicines. About 5% of adults have an episode of acute bronchitis each year. An estimated 90% of these seek medical advice for the same (Saust et al., 2018). Acute bronchitis is caused by infection of the large airways commonly due to viruses and is usually self-limiting. Bacterial infection is uncommon. Still, often antibiotics are prescribed, despite lacking effectiveness (Tanner and Karen Roddis, 2018). Most medical guidelines advice a “wait-and-see” policy, the use of antihistamines and cough medicines is discouraged.

### 1.2 Objectives

In the last decade we experienced a renaissance of herbal medicines with a rising demand especially for the treatment of the before-mentioned indications. This implicates that there is an urgent need for a scientific progress towards a rational phytotherapy, which will combine the benefits of “Modern Medicine” with the “Traditional Knowledge” on the therapeutic benefits of herbal medicines.

In order to create a basis of knowledge to build upon novel interdisciplinary research ideas towards the establishment of herbal medicines into rational therapeutic strategies, we extracted information from clinical studies. Thereby we aimed to get an overview on.

- which herbal medicines have been studied so far for which ailment
- which outcomes have been studied
- what quality level (level of evidence) the published studies have

Answering these questions, we create a comprehensive critical picture of the current knowledge on clinical efficacy and benefits as well as on failures and possible adverse events. Based on the results of these studies we give recommendations for practitioners and patients.

## 2 Methods

### 2.1 Search strategy and selection of scientific reports

Information on the therapeutic use of herbal medicines in different ailments was collected from scientifically published articles by conducting a search in the database PubMed for each of the five indication groups according to the following inclusion and exclusion criteria.

#### 2.1.1 Inclusion criteria

1. Herbal Medicine

AND

2. Disorders/complaints (see section “Indications Suitable for Treatment with Herbal Medicines”). Depending on the ailment, the term “herbal medicine” was combined with a, b, c, d, or e respectively:
  a.Psychosomatic symptoms (depressive disorder, sleeping disorders/insomnia, anxiety, cognitive impairment)
  b.Gynecological complaints (climactic symptoms, menstrual symptoms, premenstrual syndrome)
  c.Gastrointestinal disorders/dyspepsia
  d.Urinary tract infections
  e.Upper respiratory tract infections

AND

3. Clinical Trial
Exclusion criteria
a. Reports in languages other than German or English language
b. No full-text available
c. Study protocols
d. Traditional medicine (e.g., Traditional Chinese Medicine, Ayurveda, *etc.*),
e. Aroma therapy
f. Dietary supplements
g. Self-made extracts and preparations
h. Adjuvant treatment with herbal medicine
i. Herbal medicines without market access in the EU
j. *In vivo*/*in vitro* studies (pre-clinical studies)
k. Homeopathy
l. Acupuncture/acupressure
m. Children and youth (under the age of 18 years)
n. Healthy volunteers
o. Primary preventive interventions (incl. Pre-post-operative complaints)
p. Predominant comorbidities
q. Case studies/case reports
r. Televised, internet-based or web-based trials

Reasons for exclusion criteria:

a, b: Authors should be able to read and understand the full text; c: clinical results should have been obtained from a study; d, e, f, g, h, i: selected in order to filter all available information on legally approved (in Europe in particular in Germany) herbal medicines or the respective standardized extract (HMPC Monographs of the European Medical Agency - EMA) only; j: preclinical evidence should be excluded; k, l: alternative naturopathic therapy forms should be excluded; m: children should be excluded due to different drug metabolism; n, o: healthy volunteers should be excluded in order to obtain information on clinical therapeutic benefits; p: predominant comorbidities should be excluded because they can affect the efficacy of the herbal drug in particular when co-administered with other drugs; q; clinical benefits from single cases are difficult to generalize; r: excluded for methodological reasons, e.g., data interpretation.

### 2.2 Data extraction and quality assessment of scientific reports

To get an overview on the characteristics of all included articles, a table was created for each indication group containing information on the publication, the study design, the population and treatment duration, the indication and the primary outcome, the herbal medicine and comparison treatment (comparator) as well as the results. Furthermore, we performed a quality assessment of the collected reports according to the following scoring method.

• 1 point for an observational study or a pre-post observational comparison
• 2 points for a clinical trial
• 3 points for a randomized controlled trial plus 1 additional point for blinding

**FIGURE 1:**
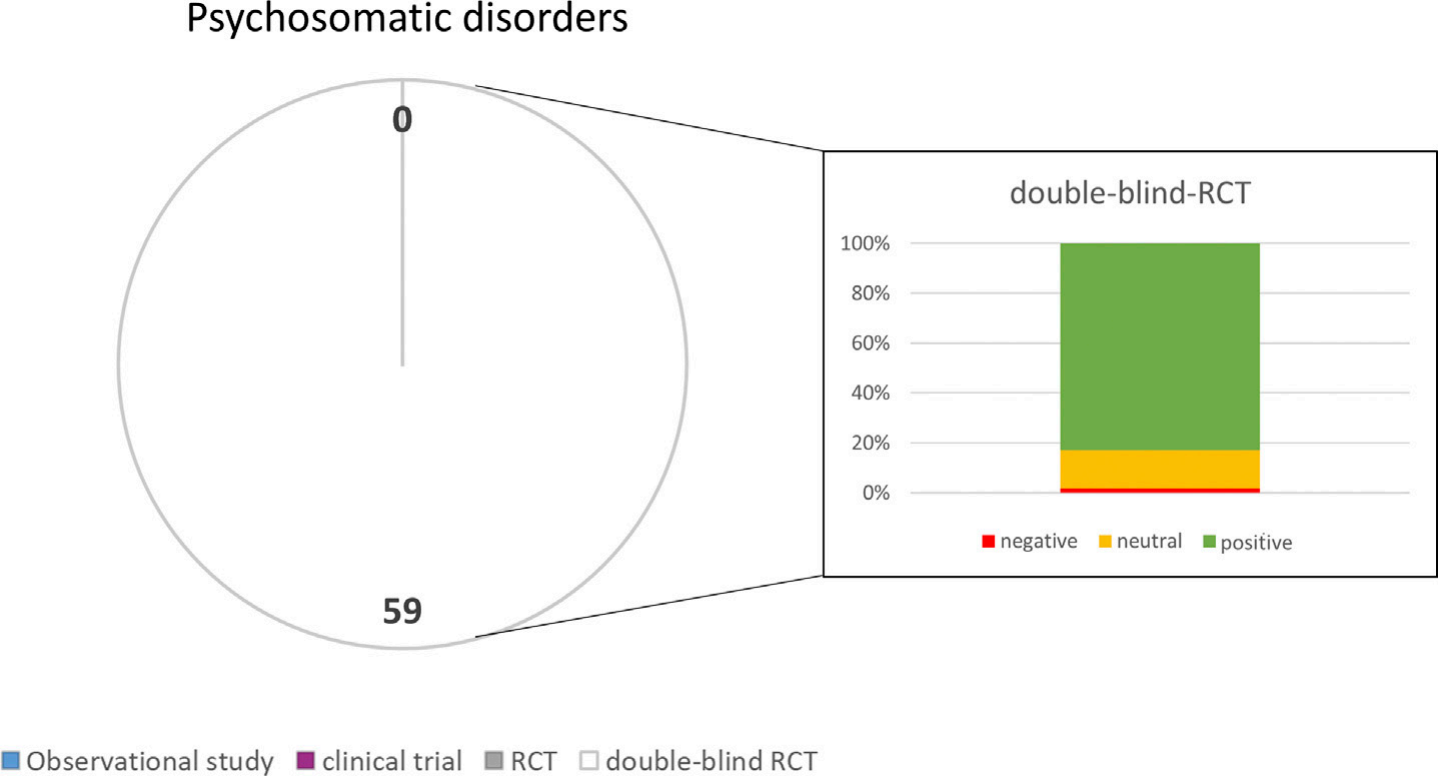
Numbers of studies and outcomes.

**FIGURE 2:**
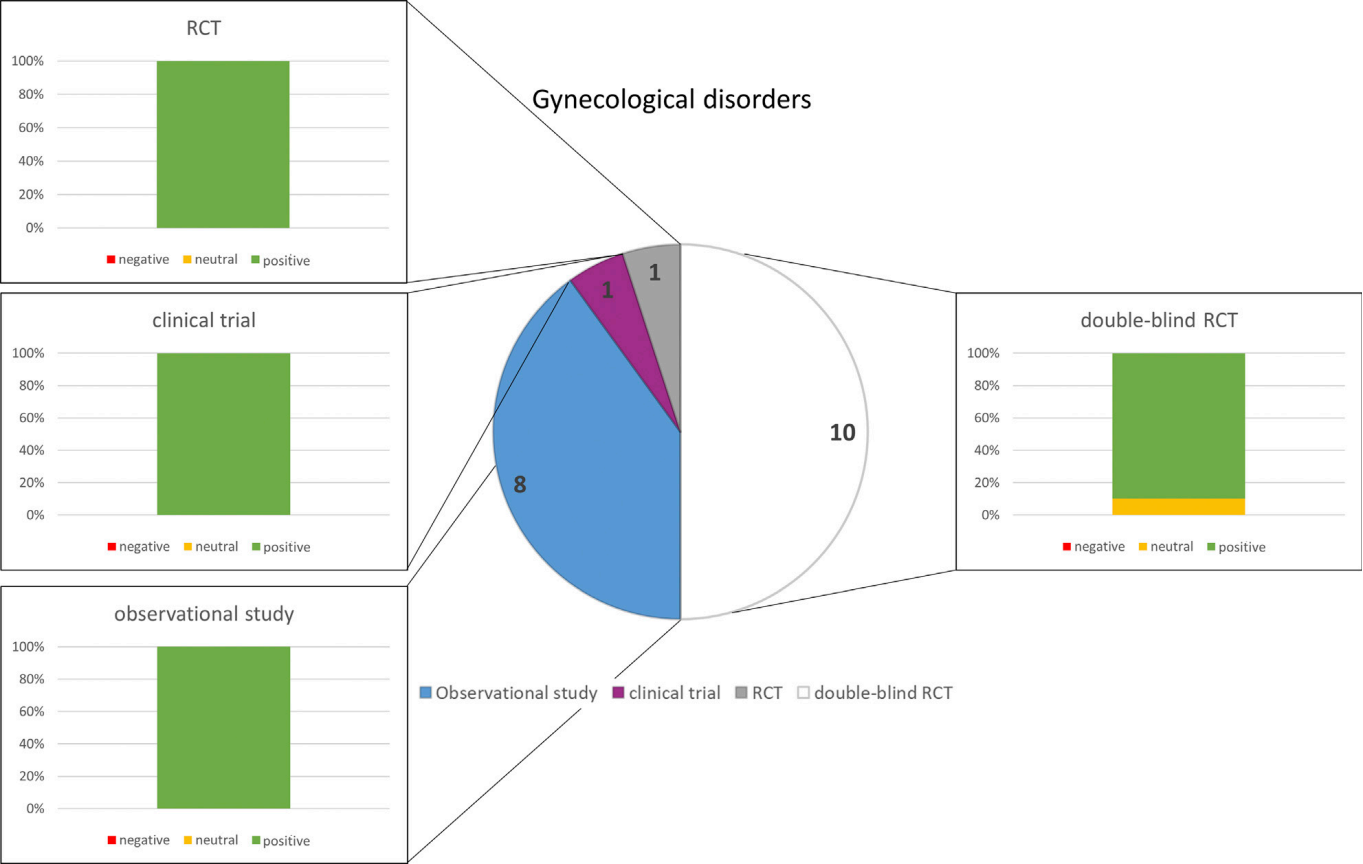
Numbers of studies and outcomes.

**FIGURE 3:**
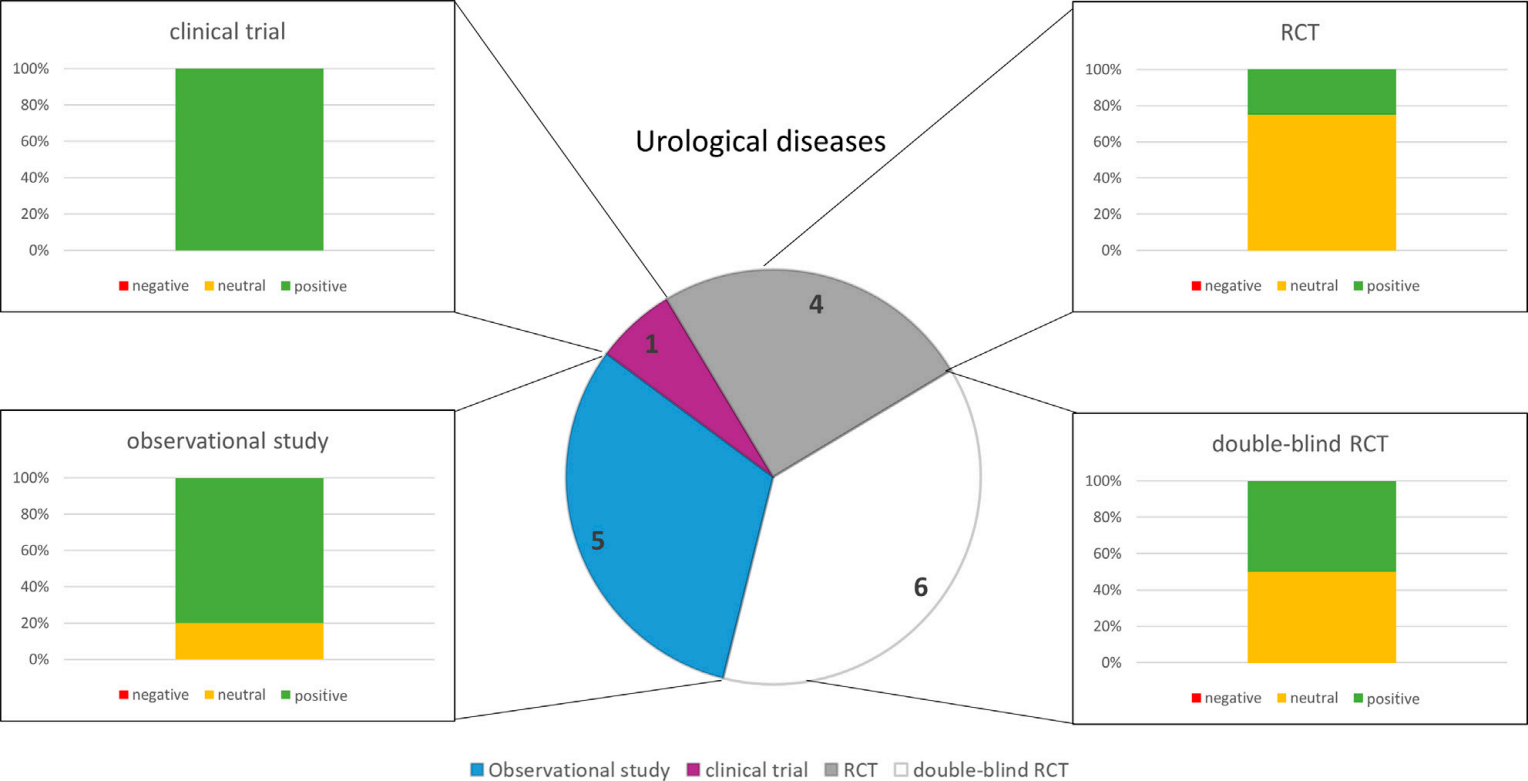
Numbers of studies and outcomes.

**FIGURE 4:**
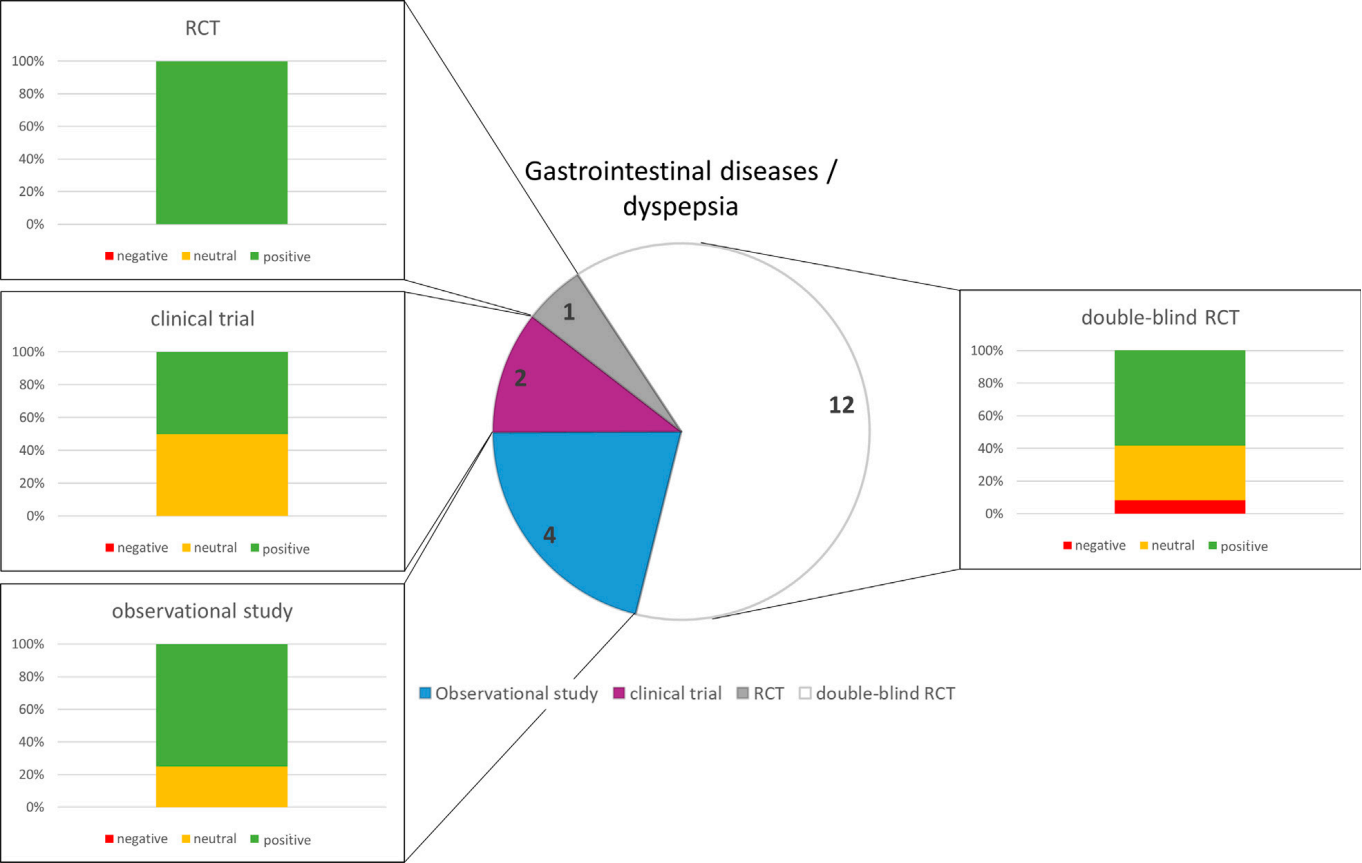
Numbers of studies and outcomes.

**FIGURE 5:**
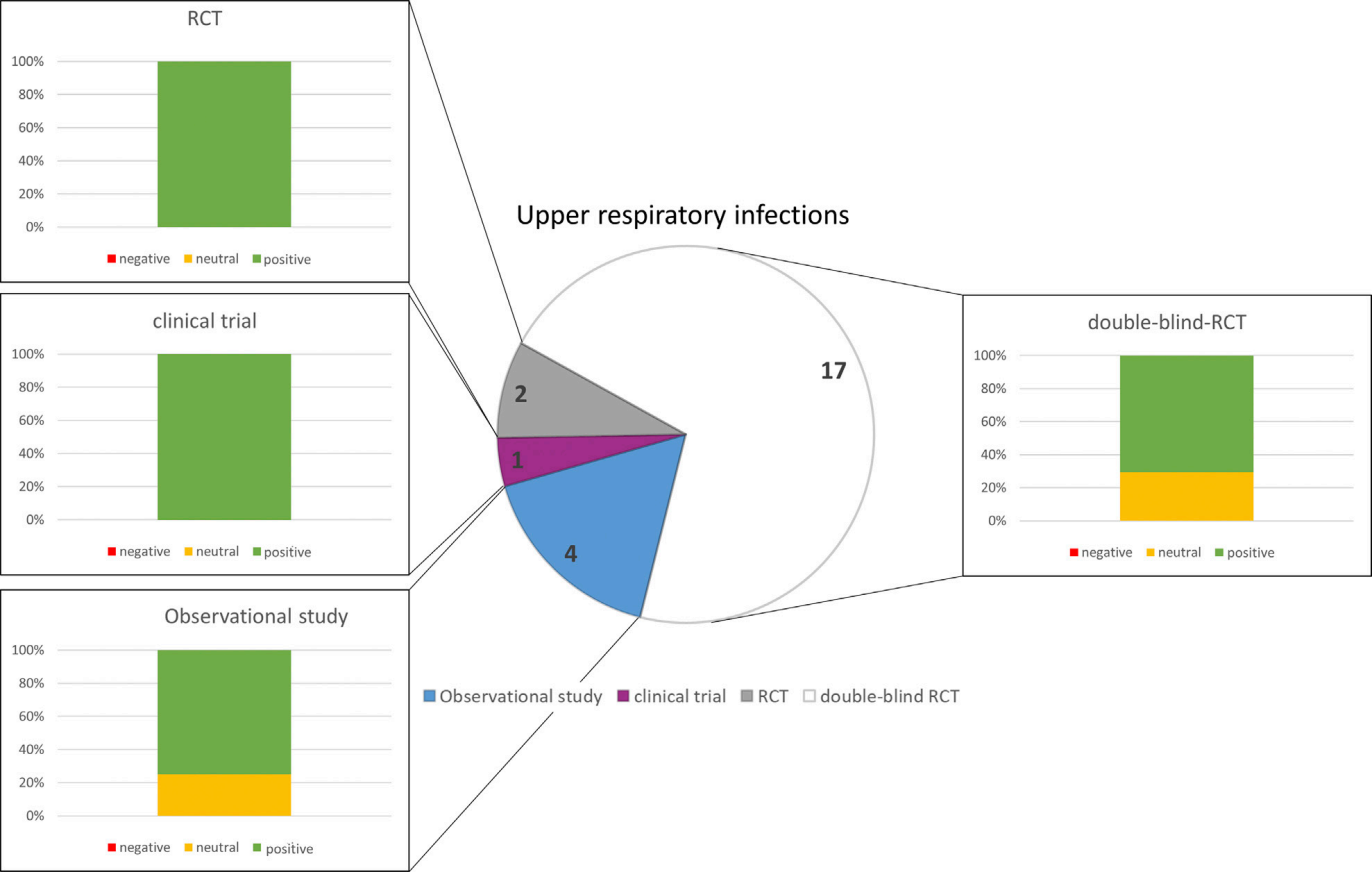
Numbers of studies and outcomes.

Thereby, a score between 1 and 4 was obtained indicating the quality for all scientific reports; respectively publications with the highest level of evidence (RCT + blinded) had a scoring value of four points (see Figures 1–5).

## 3 Results

### 3.1 Psychosomatic disorders

A search for publications with the terms “psychosomatic disorder” and “herbal medicine” yielded only 64 results. Therefore, the search was extended with more specific terms (see inclusion criteria) yielding in 4.440 hits for depressive disorder, 1.907 hits for sleeping disorders, 2.380 hits for anxiety and 1.374 hits for cognitive impairment including Alzheimer’s disease. After eliminating all publications according to the exclusion criteria 59 publications remained. Among those, 39 studies were related to depressive disorders, 4 to sleeping disorders, 6 to anxiety and 10 to cognitive impairment and Alzheimer’s disease (neurological disorders). Most of them were double blind randomized controlled trials (quality group 4). For the treatment of depressive disorders predominantly *Hypericum perforatum* L (St. John’s Wort; SJW) was used and only few studies examined the clinical benefits of *Rhodiola rosea* L (Rosewood). *Valeriana officinalis* L (Valerian Root) and *Humulus lupulus* L (Hops) extracts were preferred for the treatment of sleeping disorders, while for anxietyextracts of Lavandula angustifolia (Lavender) were studied. Extracts of *Ginkgo biloba* L (Maidenhair Tree) were used in clinical studies with patients having neurological disorders (cognitive impairment and Alzheimer’s disease). Supplementary Table S1 provides an overview of the studies, their characteristics and results (see also Figure 1).

#### 3.1.1 Depressive disorders

The use of herbal medicines in depressive disorders is well examined and in particular the clinical benefits of SJW are well supported by clinical studies of high quality. All 37 selected studies on the use of SJW in depressive disorders ranging from mild to severe forms have been double-blind randomized controlled trials (quality group 4). Study duration was predominantly between 4 and 8 weeks and only few studies examined the effects for longer time periods of up to 6 months. The majority of the studies reported positive therapeutic effects concerning Hamilton depression rating scale (HAMD) as primary outcome parameter and only 5 of them (Shelton et al., 2001; Davidson et al., 2002; Bjerkenstedt et al., 2005; Moreno et al., 2006; Rapaport et al., 2011) did not demonstrate superiority as compared to placebo or pre-post.

In six studies (published predominantly before the year 2000) comparing SJW with tricyclic anti-depressive drugs the clinical benefits of the herbal drug in respect to placebo or in pre-post comparison was at least equal to the synthetic drug no matter if it was imipramine (Vorbach et al., 1994; Vorbach et al., 1997; Philipp et al., 1999; Woelk, 2000), maprotiline (Harrer et al., 1994) or amitriptyline (Wheatley, 1997). However, with regards to tolerability, SJW was clearly superior to any of the tricyclic antidepressants.

The more recent studies compared the efficacy of SJW with the selective serotonin reuptake inhibitors (SSRI) paroxetine, sertraline, citalopram and fluoxetine. In most of the 18 studies the therapeutic benefits of SJW were at least equal to those of the SSRIs (Harrer et al., 1999; Berger et al., 2000; Brenner et al., 2000; Friede et al., 2001; van Gurp et al., 2002; Bjerkenstedt et al., 2005; Gastpar et al., 2005; Szegedi et al., 2005; Anghelescu et al., 2006; Gastpar et al., 2006; Sarris et al., 2012). In two studies SJW was even superior to fluoxetine (Fava et al., 2005) or paroxetine (Seifritz et al., 2016) in reducing depressive symptoms. In one study the responders of a previous study were included in a further RCT testing the efficacy of SJW against citalopram. Here the numbers of patients with relapse was lower in the SJW group as compared to citalopram (Singer et al., 2011). The results of one study indicated that SJW was less efficacious than both fluoxetine and placebo, however in this study the group on SJW had the lowest remission rates (Moreno et al., 2006). In two studies no statistical differences in HAMD scores between SJW, placebo and citalopram (Rapaport et al., 2011) or sertraline (Davidson et al., 2002) could be found with adverse effects in the SJW and the SSRI groups.

In most of the above-mentioned studies, comparing the efficacy of SJW to standard therapy, a placebo group was included. However, in 13 studies SJW was tested exclusively against placebo whereby two of these studies examined the efficacy of different dosages of SJW extract (Laakmann et al., 1998; Kasper et al., 2006). In these studies, the higher concentrations had the better clinical benefits. In a continuation study of the effect of SJW in long term treatment a higher dosage (1,200 mg/d) was not superior to the lower one (600 mg/d) (Kasper et al., 2007). Interestingly the higher dosages were still well tolerated although mild adverse events related to gastrointestinal disorders were observed in a small portion of the patients (Kasper et al., 2006). In only one of our selected studies SJW was not effective in comparison to placebo for the treatment of major depression but safe and well tolerated (Shelton et al., 2001). In all other studies SJW was superior to placebo no matter if given in low (Laakmann et al., 1998; Lecrubier et al., 2002; Randlov et al., 2006), medium (Kasper et al., 2006; Kasper et al., 2007; Mannel et al., 2010) or in high (Hansgen et al., 1994; Harrer et al., 1994; Sommer and Harrer, 1994; Kalb et al., 2001; Uebelhack et al., 2004; Kasper et al., 2006; Kasper et al., 2007; Kasper et al., 2008) dosages.

For the efficacy of Rhodiola rosea in treatment of depressive disorders only few studies were performed so far. Therefore, a clear conclusion cannot be drawn, especially as the outcomes are not homogenous. While one study investigating the efficacy of R. rosea against placebo and the SSRI sertraline reported on a statistically not-significant inferiority of the herbal medicine (Mao et al., 2015) another study demonstrated clinical benefits concerning the symptoms of depression, insomnia, emotional instability and somatization against placebo. In this study two dosages of R. rosea were tested and the higher dose (680 mg/d) showed even positive effects on self-esteem (Darbinyan et al., 2007).

#### 3.1.2 Sleeping disorder

Interestingly the search for qualitatively high clinical studies (according to our inclusion and exclusion criteria) revealed only few studies. The majority of them investigated the efficacy of valerian alone (Donath et al., 2000) or in combination with hops (Koetter et al., 2007) compared to placebo (Donath et al., 2000; Koetter et al., 2007) or to oxazepam (Dorn, 2000; Ziegler et al., 2002). All studies reported clinical benefits, however while the one research group reported that valerian alone was efficacious against insomnia (Donath et al., 2000) the other group reported on clinical benefits only in combination with hops (Koetter et al., 2007). Both study designs were placebo-controlled. In comparison to oxazepam valerian was not inferior and both therapy options improved sleep quality (SF-B) in a similar fashion (Dorn, 2000; Ziegler et al., 2002).

#### 3.1.3 Anxiety

Herbal Medicines with lavender extracts were clinically studied for the treatment of anxiety. Between 2010 and 2019 six qualitatively high studies performed in Germany, Austria and Switzerland reported on the beneficial effects of lavender against symptoms of anxiety with improvements on the Hamilton anxiety rating (HAMA) scale as primary outcome (Kasper et al., 2010; Woelk and Schlafke, 2010; Kasper et al., 2014; Kasper et al., 2015; Kasper et al., 2016; Seifritz et al., 2019) and all studies used the same extract (WS1265). Four of the 6 studies were performed by the same group, however the study design differed. In these studies the efficacy of lavender was either compared to placebo (Anghelescu et al., 2006; Kasper et al., 2010; Kasper et al., 2016; Seifritz et al., 2016) and/or to paroxetine (Kasper et al., 2014) and lorazepam (Woelk and Schlafke, 2010). Overall, the lavender preparation was regarded as efficacious and safe.

#### 3.1.4 Neurological disorders (cognitive impairment and Alzheimer)

We selected 10 studies investigating the efficacy of ginkgo biloba extract in the treatment of cognitive impairment and Alzheimer’s Disease (AD) with 8 of them testing against placebo (Le Bars et al., 1997; Le Bars et al., 2002; Le Bars, 2003; van Dongen et al., 2003; Schneider et al., 2005; Napryeyenko et al., 2007; Gavrilova et al., 2014; Gschwind et al., 2017), one against rivastigmine (Nasab et al., 2012) and one against donepezil (Mazza et al., 2006). In three of the studies two different ginkgo extracts did not show superiority over placebo regarding the primary outcome. In detail 5 of the studies showed that extracts of ginkgo biloba lead to a decrease in NPI composite score (Gavrilova et al., 2014) improved significantly ADAS-Gog and GERRI (Le Bars et al., 1997; Le Bars et al., 2002; Le Bars, 2003), or the SKT test battery (Napryeyenko et al., 2007) as outcome parameters. In three studies ginkgo extracts did not show superiority over placebo regarding the primary outcome parameters ADAS-cog (Schneider et al., 2005), gait analyses (Gschwind et al., 2017) or SKT test-battery (van Dongen et al., 2003), whereby in one of these studies the primary outcome parameter ADAS-cog also declined in the placebo group rendering the results of the study inconclusive (Schneider et al., 2005). With respect to the AD conventional medication rivastigmine, ginkgo biloba extract was inferior regarding the primary outcome parameters MMSE and SKT test-battery (Nasab et al., 2012). Finally one study in which gingko biloba was more efficacious than placebo and equal to the second generation cholinesterase inhibitor donepezil (Mazza et al., 2006) was heavily criticized by two other groups (Corrao et al., 2007; Korczyn, 2007), making it difficult to estimate if the use of ginkgo containing herbal medicines are justified for the treatment of mild to moderate AD.

### 3.2 Gynecological complaints

Of 383 search hits, 20 articles met the inclusion criteria. Eleven studies were related to menopausal symptoms and nine to PMS. Most were double-blind randomized controlled trials or observational studies (Figure 2). The studies on menopausal symptoms reported mainly positive results and the results concerning PMS were exclusively positive (Figure 2). The tested phytopharmaceuticals contained *Cimicifuga racemosa* (L.) (Black cohosh) (10 studies) and *Salvia officinalis* (Sage) (1 study) for the treatment of menopausal symptoms and *Vitex agnus-castus* L (VAC, Chaste tree) (8 studies) and SJW (1 study) for PMS. Supplementary Table S2 provides an overview of the study characteristics and results.

#### 3.2.1 Menopausal symptoms

In studies examining the clinical benefits of black cohosh for the treatment of menopausal symptoms, sample sizes ranged from *n* = 62 to *n* = 6,141. Treatment duration was between 12 weeks and 9 months. The herbal drug dosages ranged from 20 to 127.3 mg.

In comparison to HRT, the benefit-risk-balance points to significant non-inferiority and superiority of black cohosh (Bai et al., 2007). In three other studies menopausal complaints improved overall, but differences between black cohosh and HRT were not significant (Wuttke et al., 2003; Nappi et al., 2005; Friederichsen et al., 2020). The combination of black cohosh with SJW significantly reduced menopausal complaints and was superior to transdermal estradiol (Briese et al., 2007). Independent of a high or low dose, menopausal complaints decreased significantly (Liske et al., 2002; Drewe et al., 2013). Adverse events rates were lower in the low dose group (Drewe et al., 2013) or similar to the high dose group (Liske et al., 2002). Menopausal symptoms decreased significantly more for black cohosh compared to placebo (Osmers et al., 2005). In another study with 62 participants, the difference between the symptom scores just approached significance (Wuttke et al., 2003). Interestingly, this also applies to the comparison of conjugated estrogens and placebo. Adverse events rates did not differ significantly between black cohosh and placebo (Wuttke et al., 2003; Osmers et al., 2005). Significant and clinically relevant reductions in menopausal symptoms (Vermes et al., 2005) or higher quality of life (Julia Molla et al., 2009) were observed after treatment with black cohosh compared to therapy start. Sage taken for 8 weeks significantly decreased the number of menopausal hot flushes from week to week (Bommer et al., 2011). Observed treatment-related adverse events were mild and occurred in only one person. However, no comparison was made to another treatment or placebo.

#### 3.2.2 Premenstrual syndrome

Eight studies dealt with the treatment of PMS with VAC. The sample sizes ranged from *n* = 43 to *n* = 1,634. Treatment duration was three cycles; Berger et al. (2000) added three subsequent cycles without treatment. The administered dosages ranged from 1.6 to 20 mg extract.

Results of studies comparing VAC with pyridoxine or placebo were similar. PMS symptom reduction was significantly more pronounced for VAC compared to pyridoxine (Lauritzen et al., 1997) or placebo (Schellenberg, 2001; Bachert et al., 2009; Barrett et al., 2010; Schellenberg et al., 2012). Rates of adverse events were similar between groups in each study (Loch et al., 2000; Schellenberg, 2001; Barrett et al., 2010; Schellenberg et al., 2012). Schellenberg et al. (2012) compared a VAC reference dose to a lower and higher dose; the results were in favor for the reference dose compared to the low dose. No significant differences between the high and reference dose emerged. The number of participants with adverse events was slightly elevated for the high dose. In single-arm studies, symptoms of PMS significantly decreased after three cycles of VAC treatment (Berger et al., 2000; Loch et al., 2000; Momoeda et al., 2014). Only mild PMS-like adverse events were observed. Berger et al. demonstrated a gradual symptom return after therapy completion (Berger et al., 2000). PMS symptoms were significantly higher compared to the end of the treatment, but still 20% lower than at baseline.

A clinical study testing the efficacy of SJW in treating mild PMS (Canning et al., 2010) demonstrated significant improvements in physical (e.g., food craving) and behavioral (e.g., confusion) symptoms compared to placebo. The effect on mood (e.g., irritability) and pain (e.g., cramps) was not significant.

### 3.3 Gastrointestinal disorders

A search for publications with the search terms “gastrointestinal disorder” and “herbal medicine” yielded a total of 19 results after applying the exclusion criteria. Of these, eight studies were related to hepatic disorders, three publications dealt with IBD, two studies focused on IBS, and six studies had been done on FD. Most of them were done in a double-blinded randomized controlled manner (*n* = 12) (Figure 3). *Silybum marianum* (L.) Gaertn (Silymarin, milk thistle) was used in patients suffering from a hepatic disease. Patients with IBD were treated with Artemisia absinthium L (wormwood) or *Potentilla erecta* (tormentil). The standardized extract STW 5 containing *Iberis amara* (bitter candytuft), *Glycyrrhiza glabra* L (Liquorice), *Carum carvi* L (caraway), *Mentha* ×*piperita* (peppermint), *Melissa officinalis* L (lemon balm)*, Matricaria chamomilla* (chamomile)*, Angelica archangelica* (wild celery), *Chelidonium majus* (greater celandine) and milk thistle has been applied in IBS and FD. The same has been done with the standardized extract STW 5-II which in contrast to STW 5 is free of wild celery, greater celandine, and milk thistle. SJW has been used to treat patients suffering from IBS. A combination of the standardized extracts WS 1340 (peppermint oil) and WS 1520 (caraway oil) was used for patients with FD. Supplementary Table S3 and Figure 3 provide an overview of the study characteristics and results.

#### 3.3.1 Hepatic disease

Trials on steatohepatitis, cirrhosis and different kinds of hepatitis (*n* = 18) included patient cohorts ranging from 14 to 200 participants, all of them aged >18 years. Patients were treated with silymarin orally or intravenously (Pares et al., 1998; Tanamly et al., 2004; Ferenci et al., 2008; Hawke et al., 2010; Fried et al., 2012; Adeyemo et al., 2013; Fathalah et al., 2017; Tanwar et al., 2017) with dosages ranging from 280 to 2,100 mg/day or 5–20 mg/kg/day, respectively. Six studies compared the HM group to a placebo group (Pares et al., 1998; Tanamly et al., 2004; Hawke et al., 2010; Fried et al., 2012; Adeyemo et al., 2013; Tanwar et al., 2017). Silymarin did not reduce virus titers and/or serum alanine transaminase (ALT) in patients with Hepatitis C and non-alcoholic Steatohepatitis C, compared to placebo (Adeyemo et al., 2013). The same observation has been made by others (Hawke et al., 2010). Furthermore, the integration of silymarin into a PEGylated (Peg)-interferon based regimen did not improve the outcome of HCV patients in terms of HCV RNA suppression and Enhanced Liver Fibrosis score performance (Tanamly et al., 2004). There was also no effect of silymarin on HCV patients who were previously unsuccessfully treated with interferon (multicenter, double-blind, placebo-controlled trial) (Fried et al., 2012). Although HCV-patients reported to “feel better” after 12 months of silymarin therapy in a further study, symptoms and quality of life (QOL) scores did not differ between the silymarin and the placebo group (Tanamly et al., 2004). Treatment with silymarin was also well tolerated over a period of 2 years. However, the course of liver cirrhosis in this patient cohort has not been improved (Pares et al., 1998). Contrasting these results, dose escalating studies on HCV cirrhotic patients revealed positive effects of silymarin or silibinin (also milk thistle), in a way that high-dosed silymarin (1,050 mg/day) improved QOL and biochemical parameters of chronic HCV-decompensated cirrhotic patients with no serious adverse events (Ferenci et al., 2008; Fathalah et al., 2017) compared to low-dosed silymarin (420 mg/day). Notably, silibinin exerted a dose-dependent antiviral effect on Peg-interferon/ribavirin non-responders (Ferenci et al., 2008; Fathalah et al., 2017).

#### 3.3.2 Inflammatory bowel disease (IBD)

Between 2007 and 2009, three clinical trials on CD or IBD have been conducted, two in Germany (quality groups 1 and 2) and one in the United States (quality group 4) (Huber et al., 2007; Omer et al., 2007; Krebs et al., 2010). Patients were treated with wormwood or tormentil for 3–10 weeks. A total of 30 patients were treated with wormwood or placebo (Omer et al., 2007; Krebs et al., 2010). In this context, wormwood decreased tumor necrosis factor alpha levels and the CD activity index score, whilst scores for IBD questionnaire and Hamilton depression scale have been improved, compared to the controls (Omer et al., 2007; Krebs et al., 2010). Daily intake of tormentil reduced clinical activity index scores in all patients, however, during the wash out phase scores increased again. Tormentil has been proven to be safe for ulcerative colitis patients in dosages up to 3,000 mg/day (Huber et al., 2007).

#### 3.3.3 Irritable bowel syndrome (IBS)

Symptoms of IBS were treated with STW 5 and STW 5-II or SJW (both studies were quality group 4) (Madisch et al., 2004b; Saito et al., 2010). The clinical trial carried out by Madisch et al. compared the effects of the treatment group with those of bitter candytuft mono-extract and placebo. STW 5 and STW 5-II (60 drops/day over 4 weeks) significantly reduced the total abdominal pain and the IBS score compared to placebo and bitter candytuft mono-extract (Madisch et al., 2004b). The study carried out by Saito and others investigated the clinical efficacy of SJW pointing to a lower effect as compared to placebo (Saito et al., 2010).

#### 3.3.4 Functional dyspepsia (FD)

Six studies on patients suffering from FD were performed, including treatment with either a WS 1520/WS 1340 combination (*n* = 3) (Madisch et al., 1999; Rich et al., 2017; Storr and Stracke, 2022) or with STW 5 (von Arnim et al., 2007) and/or STW 5-II (*n* = 3) (Rösch et al., 2002; Madisch et al., 2004a). WS 1340/WS 1520 was documented to be a “valuable” (Storr and Stracke, 2022) or an “effective” therapeutic regimen (Rich et al., 2017), as it relieved pain and improved disease-specific QOL, compared to placebo. The primary outcome of WS 1340/WS 1520 was also proven to be comparable to the prokinetic agent cisapride (Madisch et al., 1999).

It is to be noted that the use of cisapride has meanwhile be restricted by the EMA due to the risk of potentially life-threatening cardiac arrhythmia [https://www.ema.europa.eu/en/medicines/human/referrals/cisapride].

Similar results have been presented in the STW 5 and STW 5-II trials. The gastrointestinal symptom score was significantly lowered when compared to the placebo group (Madisch et al., 2004a; von Arnim et al., 2007), with a therapeutic response comparable to cisapride (Rösch et al., 2002).

### 3.4 Urinary tract infection (UTI) and lower urinary tract symptoms (LUTS)

Initial search on herbal drugs in urologic clinical trials pointed to 263 manuscripts published between 1983 and 2022. Narrowing the search to “herbal medicine” (HM) 18 relevant publications were identified. One publication was nearly identical to another one and, therefore, has not been taken care of in this chapter, one article only reviewed former trials (16 publications remaining). All of them were related to lower urinary tract infection (UTI), or acute uncomplicated cystitis, respectively. Four different HM have been applied, either compared to placebo or guideline-based treatment (*n* = 12).

#### 3.4.1 Urinary tract infections (UTI)

Several studies investigated the standardized herbal extract BNO 1045 which contains *Centaurium erythraea* Rafin, herba (Centaury); Levisticum officinale Koch, radix (Lovage); and Rosmarinus officinalis L., folium (Rosemary). In two studies, the clinical benefits of BNO 1045 in preventing UTI in high-risk women undergoing urodynamic studies (UDS) (Miotla et al., 2018) or urogynecological surgeries (Wawrysiuk et al., 2022) was evaluated. High-risk women were defined as: age over 70, elevated postvoid residual urine>100 mL, recurrent UTI, pelvic organ prolapse (POP) ≥II in POP-Q scale, and neurogenic bladder. No statistical differences in UTI incidence were found between patients receiving antibiotics or BNO 1045. No superiority of antibiotics over BNO 1045 has been confirmed as well in a subsequent prospective study on postoperative UTI after midurethral sling surgery (MUS) (Rechberger et al., 2020). In another study, an herbal mixture based on D-mannose, Arctostaphylos uva-ursi, Betula pendula, and Berberis aristata was compared to BNO 1045 in reducing symptoms of UTI after MUS (Rechberger et al., 2022). The rationale was based on the EAU 2022 guidelines which recommended D-mannose as prophylaxis of UTI. In this context, BNO 1045 was proven to be similar effective, compared to the herbal mixture. The use of BNO 1045 has been documented here to be a potential and valuable alternative to antibiotics for UTI prevention. All four trials have been carried out in the same institution involving the same main investigators which were (partially) associated with the manufacturer of BNO 1045.

A randomized, double-blind, multicenter Phase III clinical trials compared the efficacy and of BNO 1045 to antibiotics concerning symptoms and recurrence rates in women with uncomplicated UTI. Based on the endpoints “UTI-recurrence” and “additional antibiotics use”, BNO 1045 was proven to be non-inferior to antibiotic treatment (Wagenlehner et al., 2018). In a retrospective cohort study, data from outpatients in Germany with at least one diagnosis of acute cystitis or UTI and a prescription of either BNO 1045 or standard antibiotics were analyzed (Holler et al., 2021). Compared to antibiotics, BNO 1045 was associated with significantly fewer recurrence rates of UTI and with reduced additional antibiotic prescription. BNO 1045 was propagated to be an effective and safe symptomatic treatment option for acute cystitis or UTI.

In an open-labeled, randomized, controlled trail the effect of BNO 1045 to prevent recurrences of cystitis in younger women was evaluated (Sabadash and Shulyak, 2017). All patients received an antibacterial therapy, the test group was additionally treated with BNO 1045. The integration of BNO 1045 prevented bacteriuria and recurrent cystitis episodes more frequently (primary outcome), compared to the control group without BNO 1045. This may indicate superiority of the combination therapy. However, interpretation of the results of the study is limited due to the lack of blinding on both sides - patients and physicians. A further study without any involvement of the manufacturer (no conflicts of interest noted) included younger women with acute uncomplicated cystitis. All patients received the same therapy, the nonsteroidal anti-inflammatory drug ketoprofen in combination with BNO 1045 (Kulchavenya, 2018). Quite interestingly, although the majority of the patients responded well to the therapy, the investigators also observed patients who only slightly responded, or did not respond to treatment at all. The authors concluded that uncomplicated cystitis might be cured by BNO 1045 instead of antibiotics which may be required only in minor cases. Still, the data seems to be over-interpreted, since patients were treated with both ketoprofen and BNO 1045 which does not allow to conclude to one drug alone.

Aside from BNO 1045, further herbal medicines have been investigated in clinical studies. Tablets with a standardized herbal extract containing *Armoraciae rusticanae* radix (Horseradish root) (80 mg) and *Tropaeoli majoris* herba (Nasturtium) (200 mg) have been applied to patients suffering from chronically recurrent UTI symptoms, with the result that recurrent UTI symptoms were less, compared to the placebo group (Albrecht et al., 2007). However, a subsequent trial failed to demonstrate non-inferiority of this extract to antibiotics due to a poor recruitment rate (Stange et al., 2017). Actually, no respective clinical trials with sufficient statistical power are underway.

#### 3.4.2 Lower urinary tract symptoms *LUTS*

Clinical studies have also been conducted with an herbal medicine containing the standardized extracts WS 1473 *Sabal serrulata* Schult.f (Sabal fruit) (160 mg) and WS1031 *Urtica dioica* L (Urtica root) (120 mg). All studies were related to the treatment of lower urinary tract symptoms (LUTS) caused by benign prostatic hyperplasia (BPH). The study protocols (placebo-controlled, double-blind, multicentric) were similar in all trials with the International Prostate Symptom Score (I-PSS), quality of life index, uroflow and sonographic parameters as the outcome measures for treatment efficacy. In one study (Lopatkin et al., 2005) patients were randomized to either the herbal medicine (WS 1473 and WS1031) (treatment group) or placebo (control group) while in another study patients received either WS 1473 and 1031 or the α1-adrenoceptor antagonist tamsulosin (Engelmann et al., 2011). A further study was based on the previous mentioned study (Lopatkin et al., 2005), whereby all patients were offered participation in a further 48-week follow-up with WS 1473/1031 (Lopatkin et al., 2007). Independent on the study design, it was concluded that WS 1473/1031 is superior to the placebo, and not inferior to tamsulosin in the treatment of LUTS. In a later re-evaluation of the data sets, WS 1473/1031 was shown to significantly improve nocturnal voiding frequency compared to placebo, with similar effects compared to tamsulosin or the 5α-reductase inhibitor finasteride (Oelke et al., 2014). No further studies have been enrolled since then. However, a database search in 2022 including 3,000 private practices in Germany revealed a significant association between WS 1473/1031 prescription and reduced incidence of urinary incontinence and urinary retention compared to tamsulosin and tamsulosin/dutasteride (5α-reductase blocker), as well as reduced incidence of erectile dysfunction compared to dutasteride (Madersbacher et al., 2023). In all four studies the manufacturer of the extract was involved.

One observational study was investigating the effectiveness of a standardized herbal extract containing a combination of *Cucurbita pepo* L (Marrow), *Rhus aromatica* bark (Fragrant sumac), and hops, in women with overactive bladder (Gauruder-Burmester et al., 2019). Of the 113 patients included, nearly the half (61 patients) used concomitant medications (e.g., antihypertensive, levothyroxine, lipid/cholesterol lowering agents, low dose ASS, NSAIDS) within the frame of a routine clinical setting. Considering the noninterventional character of this study, the herbal combination was demonstrated to improve overactive bladder symptoms and quality of life. A controlled study has not yet been initiated.

### 3.5 Upper respiratory tract infections (URTI)

The search on herbal medicines for the indication Upper Respiratory Infections revealed 24 publications.

The most common indications studied for the effectiveness of herbal medications were sinusitis, viral acute Rhisosinusitis (ARS) and common cold (N = 13), bronchitis (N = 8), and less frequently on acute cough (N = 2) and Acute lower and upper tract respiratory infections (N = 1) and chronic rhinosinusitis (N = 1). Most of them (N = 18) were double-blind randomized placebo-controlled trials, there were also randomized controlled trials that compared herbal medication to other herbal medication (N = 2) or to antibiotics (N = 1). Other study designs involved prospective cohorts (N = 3) and one retrospective cohort.

#### 3.5.1 Sinusitis/common cold and chronic rhinosinusitis

Studies on treatment of acute sinusitis and acute rhinosinusitis used a follow-up period between 7 and 14 days, with the (adapted) Sinusitis Severity Score (SSS) (N = 2), the Major Symptom Score (MSS) (N = 4), the Total Symptom Score (N = 1) and facial pain relief (N = 1) as primary endpoints. All studies reported significantly improvement of the intervention group over the placebo or control group.

The treatment of acute sinusitis and acute rhinosinusitis with Eps 7630 (standardized root extract of *Pelargonium sidoides* DC (Pelargonium) was studied in two double blind randomised placebo controlled trials (Bachert et al., 2009; Dejaco et al., 2019) and in one prospective (Perić et al., 2020), randomized, open-label, non-inferiority study comparing study medication to Amoxicillin All three studies reported a significant superiority resp. Non-inferiority for Eps 7630. The use of the standardized herbal extract BNO 1016 (*Primulae flos* (Primrose), *Gentiana lutea* Ruiz and Pav. Ex G.Don (Yellow gentian), *Rumicis herba* (Sorrel), *Sambuci flos* (Elderflower) and verbenae herba (Vervain) was tested in two randomised placebo controlled trials (Jund et al., 2015), one of which was blinded (Jund et al., 2015). Both studies showed stronger impact on the symptom score for BNO 1016 compared to placebo. One more study tested BNO 1016 in a multicenter, prospective, open-label study comparing its effect to intranasal fluticasone furoate, with patients in both groups showing improvement (Passali et al., 2015). ELOM-080 (standardized herbal drug preparation containing specially destilled oils from *Eucalyptus* (Eucalypt) and Citrus ×sinensis (Sweet orange) and *Myrtus* (Myrtle) and *Citrus limon* (L.) Osbeck (Lemon oil)) was evaluated once in a double blind randomised placebo controlled trial (Federspil et al., 1997) and once in a prospective, non-interventional parallel-group trial where the control group received BNO 1016 (Gottschlich et al., 2018). In both studies BNO 1016 showed superior results.

The use of extracts containing Echinacea for the treatment of common cold was positively tested in two studies, reporting on total number of facial tissues used in three to 7 days after intervention start (Naser et al., 2005) and on the Total Daily Symptom Scores (TDSS) after 7 days (Goel et al., 2004). No statistically significant differences were observed between treatment groups for the total symptom score (SS) after 14 days. In two other studies testing capsules/pills containing *Echinacea angustifolia* root and *Echinacea purpurea* root and *E. purpurea* herb there was no statistically significant difference between the intervention and placebo group concerning severity and duration of self-reported symptoms (Barrett et al., 2002) or global severity (Barrett et al., 2010).

In a double blind randomised placebo controlled trial BNO 1016 was tested for the treatment of chronic rhinosinusitis. The results reveal that the herbal drug was not superior over placebo regarding the Major Symptom Score (MSS) in week 8 and week 12 (Palm et al., 2017).

#### 3.5.2 Bronchitis

For bronchitis, nine studies were included, of which six were double-blind randomized placebo-controlled trials, testing EPs 7630 (N = 5) (Matthys et al., 2003; Chuchalin et al., 2005; Matthys and Heger, 2007; Matthys et al., 2010; Kähler et al., 2019) or ELOM-080 (N = 1) (Gillissen et al., 2013). The prospective observational studies included a standardized syrup of *Hedera helix* L (Ivy leaves) (N = 1) (Fazio et al., 2009), pills with ethanolic Ivy-leaves dry extracts (N = 1) (Hecker et al., 2002) and EPs 7630 (N = 1) (Matthys and Heger, 2007).

Using a follow-up period of 7 days to 4 weeks, all but one (double-blinded placebo controlled trial) (Matthys et al., 2003) reported positive effects of the study medication on either Bronchitis Severity Scores, change of symptoms and coughing frequency.

#### 3.5.3 Acute cough

The treatment of acute cough with EA-575 (standardized extract from *H. helix* L.) was tested against placebo in one double blind randomized placebo controlled trial and reported a significantly better improvement of cough severity (CS) assessed by Visual Analogue Scale (VAS) in the intervention group after 1 week as compared to placebo (Schaefer et al., 2016).

#### 3.5.4 Acute lower and upper tract respiratory infections

We included one retrospective cohort study comparing people with acute lower and upper tract respiratory infections who were prescribed a phytopharmaceutical to those who were not prescribed such drugs. They found that extract EPs 7630 (description see 3.5.1) (odds ratio (OR) 0.49 [95% CI: 0.43–0.57]) and thyme extract (OR 0.62 [0.49–0.76]) compared to no phytopharmaceutical prescription exhibited the strongest decrease in antibiotics prescriptions among patients treated by general practitioners (Martin et al., 2020).

## 4 Discussion

The aim of this review is to depict the current evidence for the therapeutic efficacy of herbal medicines. Therefore, we conducted a literature search with defined inclusion and exclusion criteria in particular to select information from clinical studies with high levels of evidence and legally approved (in Europe) herbal medicines. Certainly, life-threatening disease are not suitable for the treatment with herbal medicines. This is the reason why we limited our perspective on psychosomatic disorders, gynecological complaints, gastrointestinal disorders and common infectious diseases of the urinary and the upper respiratory tract. Additionally, we concentrated on clinical trials with adult patients. It is to be emphasized that respective studies using herbal drugs have also been done in children with psychosomatic diseases (Verlaet et al., 2017; Schloss et al., 2021), IBS (Menon et al., 2023), gastrointestinal disorders (Michael et al., 2022), UTIs (Ching, 2022), and URIs (Mancak Karakus et al., 2023) to mention only some examples.

The use of herbal medicines in the treatment of psychosomatic disorders is widespread and accordingly a high number of clinical studies was available for our analysis. In our literature search, the term “psychosomatic disorders” has been chosen. This term has not been clearly defined but is related to diseases which involve both physical and psychological illness. In other words, the respective symptoms are caused by mental processes and not directly by a physical disorder. The hits we got are based on this “terminology”. In contrast, the term “mental illnesses” which also includes psychological or behavioral manifestations is strictly defined as “health conditions with changes in emotion, thinking or behavior” (Stein et al., 2021). However, even this definition is problematic, since there are concerns about specific conditions, the discrimination between independent biological entities or value-laden social constructs, and the defined indicators of dysfunction (Stein et al., 2021). Independent on these concerns, we did not apply this search term. Therefore, we cannot exclude that (very few) articles have not been discovered with our search strategy.

For the treatment of depressive disorders, St. John’s wort is well-established and the studies we selected were predominantly positive regarding improvement of symptoms. Concurrently, SJW is well tolerated and in the majority of the studies at least equal to conventional medication like tricyclic anti-depressants and selective serotonin reuptake inhibitors, which exhibit in part notable adverse events impacting patients’ quality of life of (Voican et al., 2014; Jakobsen et al., 2017).

In contrast evidence for insomnia and anxiety was thinner. It would be worthwhile to study the use of herbal drugs as alternative medication for the treatment of sleeping disorders, as for elderly people or long term use conventional hypnotics are not always the best option (Wortelboer et al., 2002; Cheng et al., 2020). All the studies we included were using valerian root extract alone or in combination with Humulus lupulus extract and showed positive effects on sleep without notable side effects. The few studies we selected for anxiety demonstrated efficacy of lavender extract (Lavandula angustifolia) and also here we had a homogenous picture of good efficacy along with good tolerability.

Several years ago, consistent beneficial effects of Ginkgo biloba for patients with cerebral insufficiency were proven in a systematic review (Kleijnen and Knipschild, 1992). However, the methodologic quality of many trials was considered to be poor. Moreover, the studies entailed a heterogeneous collection of target health problems, ranging from overt dementia to noncognitive manifestations of brain dysfunction, such as vertigo and tinnitus. More recently, the results of several new Ginkgo biloba trials have been published, most of them focusing on dementia, and showing positive effects. Probably the most talked about is the trial of the North American EGb Study Group, which was published in the JAMA in 1997 and showed a modest improvement of the cognitive performance and the social functioning of the demented patients involved (Le Bars et al., 1997), which is well in line with the studies we have collected.

In addition, menopausal symptoms and premenstrual syndrome are suitable for treatment with herbal medicines. In the here collected studies, no overall negative effects were observed and adverse events did not occur more frequently than in the comparison groups. A consistent picture emerged when comparing herbal treatment with synthetic drugs or placebo: while herbal drugs and treatment with, e.g., HRT or pyridoxine showed equal efficacy, herbal treatment was in general superior to placebo administration, except for one study.

Effective treatment of menopausal symptoms with black cohosh is supported with multiple study designs. Regardless of the study quality, there are no contradictory results.

The evidence for the treatment of PMS with VAC initially appears similar to that of black cohosh for menopausal symptoms. However, the sample sizes have been insufficient and there was a complete lack of comparisons of VAC with other therapies. Also of interest are the hints on the importance of the dose and continuous administration. A higher dosage did not have a higher efficacy compared to the standard dosage, but slightly more participants experienced adverse events (Momoeda et al., 2014). This suggests a preference for the standard dosage of VAC. Continuous use of VAC is recommended, as it has been shown that symptoms increase significantly, even if they are still lower than before therapy (Bachert et al., 2009).

However, further research is needed for both gynecological indications. Only one study each on sage for menopausal symptoms and SJW for premenstrual symptoms was found (Lauritzen et al., 1997; Lauritzen et al., 1997; Adeyemo et al., 2013). The trend-setting results point to positive effects which have to be confirmed.

For gastrointestinal disorders herbal drugs were, at least partially, shown to be similar efficacious as the standard treatment. Selected, non-toxic plant derived natural compounds may, therefore, replace synthesized drugs which are associated with undesired negative side effects and the therapeutic potential of the compounds may depend on both the plant extract and the type of disease to be treated. Indeed, SJW was not efficacious in treating IBS, whereas WS 1340/WS 1520 and STW 5 and STW 5-II showed efficacy in both IBS and FD. Considering the broad spectrum of gastrointestinal complaints, therapy of severe liver disease may require more effort than treatment of moderate dyspepsia and, hence, herbal medicine may not replace standard therapy.

As no standard therapy has so far been established for FD (Madisch et al., 2018) and IBS (Lacy et al., 2021) the design of clinical studies is difficult, making it impossible to compare the phytodrug group with a “reference” cohort, and to finally assess the value of the phytodrugs.

Particular attention should be given to STW 5 containing greater celandine which has been related to liver and biliary tract disorders (Zielińska et al., 2018). Therefore, careful preclinical examination of potential toxic properties of a compound of question is necessary before starting clinical trials.

Overall, most of the studies were well designed (multicenter, double-blind, placebo-controlled trials) with large cohorts. Considering the low side effects and often significant improvements, it might be useful to conduct further studies to either gain more detailed information about herbal medicine or to transfer the knowledge to diseases with a similar cluster of symptoms, so that distinct ailments might particularly benefit from herbal medicine (Chey et al., 2015).

With respect to urinary tract infections (UTI), herbal medicines have been proven to be similar effective as antibiotics. Undoubtedly, the data encourages further research on herbal medicines as alternatives to antibiotics in acute lower uncomplicated UTI (Wagenlehner et al., 2018). The use of herbal medicines has also been considered to be a good and safe alternative to perioperative antibiotic prophylaxis (Miotla et al., 2018). However, whether herbal medicines may reduce or even replace antibiotics in future guideline-based regimen requires more prospective studies conducted on large groups of participants (Wawrysiuk et al., 2022).

It is important to note in this context that one study discriminated between HM responders and non-responders (Kulchavenya, 2018). This phenomenon is highly important, since it indicates that the application of HM in general might be restricted to a subset of patients. Unfortunately, no ongoing trials have been enrolled in this matter, and none of the publications cited here discussed the problem of acquired or innate resistance, at least from a theoretical point of view.

LUTS caused by BPH was treated differently than UTI, since the complications of BPH, namely, urinary incontinence, polyuria, urinary retention, and erectile dysfunction, have to be targeted. The clinical trials published so far point to the benefit of herbal medicines in reducing BPH symptoms. However, it is not clear yet whether the integration of herbal medicines may allow to reduce or even to avoid the use of standard medical therapeutics in this case.

Overall, several clinical studies conducted in the last years document a beneficial role of herbal medicines in the treatment of UTI and LUTS.

Upper Respiratory Infections (URIs) are a frequent cause of troublesome symptoms, that might be appropriately treated with herbal medicine. Most studies included in this paper evaluated herbal medicines for the treatment of acute bronchitis or common cold and acute sinusitis or rhinosinusitis.

The majority of the studies we included for the treatment of acute bronchitis tested *P. sidoides* against placebo and reported a statistically significant decrease of bronchitis symptoms and/severity. This is in line with the results of a systematic review and meta-analysis (Agbabiaka et al., 2008), although a more recent systematic review judged that the evidence was of low quality (Timmer et al., 2013). Evidence for other herbal medicines in the treatment of acute bronchitis was scarce.

For the treatment of common cold we found some indications of effectiveness of *P. sidoides*, Eucalyptus, sweet orange, myrtle and lemon oil (ELOM-080) and for Gentianae radix, Primulae flos, Sambuci flos, Rumicis herba and verbenae herba (BNO 1016). A recent systematic review with network meta-analysis, showed very little solid evidence of herbal medicine *versus* placebo for common cold, with only *P. sidoides* and *Andrographis paniculata* showing a reliable decrease of symptoms. Better results were found for herbal medicine *versus* placebo concerning health related quality of life (HRQoL) (in particular *Spicae aetheroleum*) and for symptoms (Cineole and *P. sidoides*) (Hoang et al., 2023). A further systematic review reported on the efficacy of *P. sidoides* (liquid and tablet preparation) for the treatment of acute bronchitis, showing a positive results with, however, low evidence quality (Timmer et al., 2013).

Although herbal medicines are considered to be safe in principle, this might not always be the case. Some herbal compounds are suspected to be carcinogenic and/or hepatotoxic. Herbal products have also been shown to inhibit and/or induce drug-metabolizing enzymes (Moreira et al., 2014). This has to be taken into account, since herbal medicines are often used in combination with conventional drugs. In this context, preparations with SJW may reduce the efficacy of chemotherapy and of anticoagulants but enhance the one of certain consciousness-lowering agents (e.g., sedative medicines, antidepressants) (Nicolussi et al., 2020; Scholz et al., 2021). Due to potential liver toxicity of chelidonium majus, preparations containing more than 2.5 mg daily dose of whole chelidonium alkaloids had to be withdrawn, and for all preparations with lower daily doses, their instruction leaflet must include warnings on liver toxicity (Rosien, 2019). Therefore, the drug’s safety must always be carefully investigated and guaranteed by the producers and the regulatory authorities.

The analysis of the outcomes in the selected disorders reflects that herbal medicines are most efficacious for the treatment of URTI (Figure 5), followed by gynecological complaints (Figure 2) and psychosomatic disorders (Figure 1). For the treatment of urological diseases (Figure 4) in particular UTI and LUTS, we could select only 16 studies according to our strict inclusion/exclusion criteria and therefore more studies of high quality have to be performed to gain a better insight into the efficacy of herbal drugs for these ailments. Gastrointestinal diseases hold a special position as only the added value of the phytodrugs to the conventional therapy was tested. In addition, the number of studies we selected was small (Figure 3), making it difficult to judge the efficacy of herbal drugs for this indication.

This report on the current state of research on the clinical benefits of herbal medicines for non-life-threatening ailments has some limitations.

1. The literature search had to be restricted to Pubmed, because other relevant databases like e.g., EMBASE or CINAHL have not been accessible to the authors.
2. Further limitations are the small cohorts in some of the studies
3. Or that the results/outcomes of some studies have been re-analyzed from previous studies.
4. A general obstacle of data interpretation is that for some indications, in particular for gastrointestinal diseases, herbal medicines are predominantly co-administered with standard therapy, which makes it difficult to estimate the clinical benefit of the phytodrug alone.

## 5 Perspective

Our literature research gives insights into applied herbal medicines for selected indications, the study outcomes and their quality. Based on our results, we (the authors) provide an overview for patients and healthcare practitioners which extracts can be recommended for the treatment if which disorder/complaint (Supplementary Table S1).

In this context we recommend in particular *H. perforatum* L. for depressive disorder, *V. agnus castus* L. for menstrual complaints, *Cimicifica racemose* (L.) for menopausal symptoms, a combination of *I. amara* L., *M. chamomilla* L., *Mentha* ×*piperita* L., *C. carvi* L., *G. glabra* L. and *M. officinalis* L., for functional dyspepsia, a combination of *C. erythraea*, *Levisticum officinale* W.D.J.Koch and *Rosmarinus officinalis* L. for uncomlicated urinary tract infections, *P. sidoides* DC. for bronchitis and sinusitis and finally *H. helix* for cough (Supplementary Table S1). These recommendations are based on studies with the highest levels of evidence (RCTs).

However, evidence for efficacy of herbal medicines is still not satisfying in order to integrate them in conventional medicine guidelines and standard treatment regimen, which is the reason why statutory health insurances do not reimburse the costs. In fact, herbal medicines are highly popular and accepted among patients, since their application is safe since they do not exert severe side-effects. Especially when conventional medical therapies fail due to undesired side effects having a negative impact on the quality of life, patients are willing to purchase herbal medicines at their own expense. Often doctors do not know about the self-medication activities of their patients and in consequence cannot monitor the treatment with herbal medicines and possible interactions with other drugs.

The discrepancy between available results from clinical research and the use of herbal medicines under everyday conditions shows that we need to perform more interdisciplinary research studies in the future in order to collect scientific sound evidence on their benefits. Clinical research can provide information on the efficacy of phytodrugs and the importance of genetic dispositions and metabolism as well as possible interactions with other medicines. For effectiveness under everyday conditions (from bedside to practice), methods of health services research are necessary. With the help of these, the outcomes of herbal medicines can be recorded from different perspectives, in particular those of the patients (patient-reported outcomes (PROs)). For longitudinal observations, analyses of health insurance and sales volume data are also relevant, using prescriptions and the over-the-counter sales to get a picture on the needs of the patients and the acceptance of phytotherapy by healthcare practitioners. In order to pave the way for the integration of herbal medicines into therapy guidelines and regimens, findings from clinical studies should be carefully evaluated for their transferability to everyday healthcare within the scope of health services research. This way could lead to novel rational efficacious therapy strategies with less side-effects and better compliance of the patients.

## Figures and Tables

**Table:** 

**AAMI**	Age-Associated Memory Impairment
**AD**	Alzheimer’s Disease
**ADAS-Cog**	Alzheimer’s Disease Assessment Scale-Cognitive Subscale
**ADCS-CGIC**	Alzheimer’s Disease Cooperative Study-Clinical Global Impression Of Change
**ADR**	Adverse Drug Reaction
**AE**	Adverse Effects
**ADAS-Gog**	Alzheimer’s Disease Assessment Scale-Cognitive Subscale
**ALT**	Alanine Transaminase
**ARS**	Acute Rhisosinusitis
**BDI**	Beck-Depressions-Inventar
**BEB**	Complaint Inventory for symptoms of depression („Beschwerdeerfassungsbogen”)
**B-L/BfS**	Von Zerssen’s Adjective Mood Scale
**BPH**	Benign Prostatic Hyperplasia
**BSS**	Bowel Symptome Score
**BSS**	Bronchitis Symptom Score
**CAI**	Clinical Activity Index
**CD**	Crohn’s Disease
**CDAI**	Crohn’s Disease Activity Index
**CG**	Control Group
**CGI**	Clinical Global Impression
**CGI-I**	Clinical Global Impression of Improvement
**CGI-S**	Clinical Global Impression of Severity
**CS**	Cough Severity
**DDS**	Dyspeptic Discomfort Score
**DSM-III-R**	Diagnostic and Statistical Manual of Mental Disorders, Third Edition, Revised
**DSR**	Daily Symptom Report
**ELF**	Enhanced Liver Fibrosis
**EPS**	Epigastric Pain Syndrome
**EMA**	European Medicines Agency
**FAS**	Full Analysis Set
**FD**	Functional Dyspepsia
**GAD**	Generalized Anxiety Disorder
**GAD**	General Alzheimer’s Disease
**GAF**	Global Assessment of Functioning
**GERRI**	Geriatric Evaluation by Relative’s Rating Instrument
**GIS**	Gastrointestinal Symptom Score
**GPA**	Global Patient’s Self-Assessment
**HAMA**	Hamilton Rating Scale for Anxiety
**HAMD**	Hamilton Rating Scale for Depression
**HM**	Herbal Medicine
**HRQoL**	Health Related Quality of Life
**HRT**	Hormone Replacement Therapy
**HVC**	Viral Hepatitis C
**HVB**	Viral Hepatitis B
**IBDQ**	Inflammatory Bowel Disease
**IBS**	Irritable Bowel Syndrome
**ICIQ**	International consultation on incontinence modular questionnaire
**IDS-C**	Inventory of Depressive Symptomatology- Clinician-Rated
**IDS-SR**	Inventory of Depressive Symptomatology- Self-Report
**I-PSS**	International Prostate Symptom Score
**IBD**	Inflammatory Bowel Disease
**IBS**	Irritable Bowel Syndrome
**IG**	Intervention Group
**KMI**	Kupperman Menopause Index
**LAD**	Left Alzheimer’s Disease
**LUTI**	Lower Urinary Tract Infection
**LUTS**	Lower Urinary Tract Symptoms
**MADRS**	Montgomery-Asberg Depression Rating Scale
**MCI**	Mild Cognitive Impairment
**MHT**	Menopausal Hormone Therapy
**MMDQ**	Moos‘ Menstrual Distress Questionnaire
**MMSE**	Mini-Mental State Examination
**MOS**	Mean Opinion Score
**MRS**	Menopausal Rating Score
**MSS**	Major Symptom Score
**MUS**	Mid-Urethral Sling
**NAA**	Nuremberg Gerontopsychological Rating Scale for Activities of Daily Living
**NAI**	Nuremberg Gerontopsychological Inventory
**NDI**	Nepean Dyspepsia Index
**NPI**	Neuropsychiatric Inventory
**NSAID**	non-steroidal anti-inflammatory drug
**OAB**	Overactive Bladder
**OR**	Odds Ratio
**PDS**	Postprandial Distress Syndrome
**PEG**	Polyethylene glycol
**PHF**	Pressure, Heaviness and Fullness
**PHQ-9**	Patient Health Questionnaire 9
**PMS**	Premenstrual Syndrome
**PMSD**	Premenstrual Syndrome Diary
**PMTS**	Premenstrual Tension Syndrome
**POP**	Pelvic Organ Prolapse
**PPI**	Proton Pump Inhibitors
**PRO**	Patient-Reported Outcome
**PSQI**	Pittsburgh Sleep Quality Index
**PSWQ-PW**	Penn State Worry Questionnaire
**PSYCHE**	Psychiatry Educator
**QOL**	Quality of Life
**Q-LES-Q**	Quality of Life Enjoyment and Satisfaction Questionnaire
**RAD**	Right Alzheimer’s Disease
**RCT**	Randomized Controlled Trials
**SAS**	Self-Rating Anxiety Scale
**SCL-58**	Symptom Check List- 58
**SF-36**	Short Form 36 Health Survey
**SF-B**	Sleep Questionnaire B
**SKT**	german: Syndrom-Kurztest, Cognitive Test Battery
**SS**	Symptom Score
**SSRI**	Selective Serotonin Reuptake Inhibitors
**SSS**	Sinusitis Severity Score
**SWS**	Slow-Wave Sleep
**TDSS**	Total Daily Symptom Score
**TIB**	Time In Bed
**TSIRHF**	Total Score of the mean number of Intensity-Rated Hot Flushes
**TSS**	Total Symptom Score
**UDS**	Urodynamic Studies
**URTI**	Upper Respiratory Tract Infections
**UTI**	Urinary Tract Infections
**VaD**	Vascular Dementia
**VAS**	Visual Analogue Scale
**WBS**	Wellbeing Scale

## Data Availability

The original contributions presented in the study are included in the article/Supplementary Material, further inquiries can be directed to the corresponding author.
