# Supplementary material for: Current state of research on the clinical benefits of herbal medicines for non-life-threatening ailments
Source: Front Pharmacol. 2023 Sep 28;14:1234701. doi: 10.3389/fphar.2023.1234701 (PMC10569491; doi:10.3389/fphar.2023.1234701)
Supplement: Supplementary file 1 [file Table1.docx]

| **Psychosomatic Disorders** | | | | | | | | | |
| --- | --- | --- | --- | --- | --- | --- | --- | --- | --- |
| Authors | Year of publication / Journal | Country | Study score | Study design | Population (N) / Duration | Indication / Outcome | Treatment | Comparison | Results |
| **Depressive disorder** | | | | | | | | | |
| St John’s worth | | | | | | | | | |
| Laakmann et al. | 1998  Pharmaco-psychiatry | Germany | 4 | randomized, double-blind, placebo-controlled, multicenter study, | 147/ 42 days | Mild to moderate depression / HAMD, CGI, D-S | two extracts of SJW extract WS 5573 (300 mg, with a content of 0.5% hyperforin), or WS 5572 (300 mg, with a content of 5% hyperforin) or placebo | Placebo | with WS 5572 largest HAMD reduction versus day 0 followed by WS 5573 and the placebo  WS 5572 was superior to placebo in alleviating depressive symptoms.  2/3 of patients no adverse effects; Incidence of adverse effects lowest in WS 5572 group; most frequently reported impairment: headache |
| Mannel et al | 2010  Psychiatr. Res. | Germany | 4 | double-blind, randomized, and placebo-controlled trial | 200 / 8 weeks | Atypical depression (mild, moderate and severe depression) / HAMD, HAMA, PHQ-9, CGI-I | SJW (LI160)  SJW 600 mg or placebo | Placebo | highly significant benefit of LI160 for moderately depressed patients regarding primary outcome (HAMD); Secondary outcome parameters HAM-A, PHQ-9, and CGI-I scales were superior for LI160 (p<0.01).  side effects: equal tolerability and safety of LI160 and placebo, no serious side effects |
| Shelton et al. | 2001  JAMA | USA | 4 | Randomized, double-blind, placebo-controlled clinical trial | 200 / 4 – 8 weeks | Major depression / HAMD, HAMA, CGI-S, CGI-I, GAF, BDI | SJW extract  SJW 900 mg/d for 4 weeks, increased to 1200 mg/d in the absence of an adequate response thereafter) or placebo for 8 weeks. | Placebo | SJW not superior to placebo for treatment of major depression  Side effects: only headache more frequent in SJW group vs placebo (39/95 [41%] vs 25/100 [25%], respectively). |
| Uebelhack et al. | 2004  Advances in Therapy | Germany | 4 | double-blind, randomized, placebo-controlled clinical trial | 140 / 6 weeks | moderate depression / HAMD, von Zerssen's Adjective Mood Scale, CGI, | SJW (STW3-VI)  SJW 900 mg / d or placebo | Placebo | HAMD score decreased significantly from baseline by approximately 11.1 +/- 4.5 points in SJW group and by approximately 3.4 +/- 3.9 points in the placebo group; additional responder analysis (Zerssen's Adjective Mood Scale, CGI, ) in favour of SJW  conclusion: SJW (STW 3-VI) once-daily (900 mg) effective and well-tolerated option for patients with moderate depressive disorders;  safety assessment: no differences between the SJW extract and the placebo |
| Kasper et al. | 2006  BMC Medicine | Austria / Germany | 4 | double-blind, randomized, placebo-controlled, multi-center clinical trial | 332 / 6 Weeks | Mild-moderate major depressive episode / HAMD, CGI, MADRS, BDI, SF-36, | SJW Extract (WS 5570)  SJW 600 mg/d or SJW 1200 mg/d or placebo | Placebo | decrease in HAMD total scores more pronounced for SJW than for placebo groups. Secondary outcome parameters showed that SJW was statistically superior to placebo; treatment response and remission better in SJW groups compared to placebo;  incidence of adverse events low in all groups |
| Kasper et al. | 2008  European Neuropsycho-pharmacology | Austria / Germany | 4 | double-blind, placebo controlled multicenter trial | 426 / 6 weeks (all) - 26 weeks (responders) | acute episode of moderate depression / HAMD, BDI, CGI | SJW extract (WS 5570)  SJW 900 mg/d or placebo | Placebo | relapse rates during continuation treatment lower for SJW than for placebo  No differences in time to relapse between SJW and placebo group  Patients treated with SJW showed more favourable HAMD and BDI time courses and CGI;  long-term treatment had a pronounced prophylactic effect of SJW (patients with early onset of depression and those with a high degree of chronicity)  Adverse event rates under SJW were comparable to placebo. |
| Lecrubier et al. | 2002  American Journal of Psychiatry | France | 4 | double-blind, randomized, placebo-controlled tria | 375 / 6 weeks | moderate major depression / HAMD, MADRS, SCL-58, CGI | SJW extract (WS 5570)  SJW 300 mg/d or placebo | Placebo | Compared to placebo, SJW group showed significantly greater reduction in HAMD scale and better remission.  placebo and SJW groups had comparable adverse events. |
| Randlov et al. | 2006  Phytomedicine | Denmark | 4 | randomized double-blind placebo-controlled study | 150 / 6 weeks | mild or moderately severe depressed episodes or with dysthymia / HAMD, BDI, VAS, D-2-test | SJW extract (PM235)  SJW mg lower (0.12% hypericine) 270 mg/d or SJW higher (0.18% hypericine) 270 mg/d or placebo | Placebo | large discrepancy in response between dysthymic and non-dysthymics  non-dysthymics were more sensitive to SJW (HAM-D)  BDI criteria better (p=0.045) for both doses of SJW compared to placebo.  VAS results better for non-dysthymic patients treated with SJW |
| Kalb et al. | 2001  Pharmaco-psychiatry | Germany | 4 | double-blind, placebo-controlled multicenter clinical trial | 72 / 42 days | Mild to moderate depression / HAMD, D-S; CGI, GPA | SJW extract (WS 5572  SJW 900 mg/d or placebo. | Placebo | Group differences in favor of SJW at days 28 and day 42.  Pre/Post comparison: HAMD decreased from 19.7 +/- 3.4 to 8.9 +/- 4.3 points in the SJW group and from 20.1 +/- 2.6 to 14.4 +/- 6.8 points in the placebo group (mean +/- SD). Responder rates were consistently higher in the SJW group. Comparable group differences in favor of SJW were also found D-S, CGI, and GPA.  Tolerability was very good in both groups. |
| Hubner et al. | 1994  J Geriatr Psychiatry Neurol | Germany | 4 | Randomized double-blind comparative study | 39 / 4 weeks | depression with somatic symptoms / HAMD, von Zerssen’s (B-L). CGI | SJW extract (LI 160)  SJW 900 mg/d or placebo | Placebo | significant improvement in the SJW group as compared to placebo. 70% of the patients treated with SJW extract were free of symptoms after 4 weeks.  No undesirable side effects were observed. |
| Kasper et al. | 2007  Wien Med Wochenschr | Austria | 4 | Randomized, controlled, double-blind study | 161 / 4 months | Mild to moderate depression / HAMD, D-S, | SJW extract (WS 5570)  600 mg/day, 1200 mg/day or placebo | Placebo | Continuation treatment after recovery; Both SJW groups showed decrease of the HAMD total score by 0.8 (600 mg /day) and 0.4 (1200 mg /day) points during treatment phase, while patients in the placebo group deteriorated by 2.1 points.  adverse events low in all therapy groups |
| Hansgen et al. | 1994  J Geriatr Psychiatry Neurol | Germany / Switzer-land | 4 | Multi-center, double-blind, randomized controlled trial | 72 / 6 weeks | major depression in accordance with DSM-III-R / BEB, CGI | SJW extract (LI 160)  SJW 900 mg/d or placebo | Placebo | significant improvement in all four psychometric tests in the SJW group as compared to the placebo group. After switching the placebo group to SJW treatment, significant improvements were found in the original placebo group.  No serious side effects were observed. |
| Sommer & Harrer | 1994  J Geriatr Psychiatry Neurol | Austria | 4 | Randomized, multi-center, double-blind, controlled trial | 105 / 4 weeks | Mild depression / HAMD | SJW extract  SJW 900 mg/d or placebo. | Placebo | HAMD reduction from 15.8 to 9.6 or 7.2 in the SJW group, and in the placebo group, from 15.8 to 12.3 and 11.3. The differences between SJW and placebo groups were statistically significant; Notable side effects were not found. |
| Sarris et al. | 2012  Pharmaco-psychiatry | Australia | 4 | randomized, double-blind, controlled study | 124 SJW; responders (8 weeks) continuation therapy until week 26 | Major depressive disorder / HAMD, BDI, CGI-S, GAF, CGI-I | SJW extract (LI 160)  SJW 900-1500 mg / d or sertraline 50 - 100 mg/d or placebo | Sertraline  Placebo | significant favourable effect only for time regarding HAMD in SJW group (p=0.036) in comparison to sertraline or placebo  Remission rates (HAM-D ≤ 7) at 26 weeks were 58 % for sertraline, 63 % for SJW and 74 % for placebo, while relapse rates (HAM-D ≥ 14) were also not significantly different between groups [sertraline = 5 (16 %); SJW = 2 (8 %); placebo = 3 (11 %)]  Sertraline significantly increased rates of diarrhea, nausea, sweating, and anorgasmia compared to placebo |
| Van Gurp et al. | 2002  Can Fam Physician | Canada | 4 | Double-blind, randomized, controlled trial | 87 / 12 weeks | Depressive symptoms / HAMD, BDI | SJW (900 to 1800 mg/d) or sertraline (50 to 100 mg/d) | Sertraline | No important differences in mean HAMD and BDI scores (using intention-to-treat analysis), with and without adjustment for baseline demographic characteristics, between the two groups at 12 weeks.  more side effects in sertraline group than in SJW group with more benign side effects of SJW |
| Gastpar et al. | 2005  Pharmaco-psychiatry | Germany | 4 | randomized, controlled, double-blind trial | 241 / 6 months | moderate depressive disorder / HAMD, BfS, CGI | SJW extract (STW3-VI)  SJW 600 mg/d or sertraline 50 mg/d | Sertraline | After the first 12-weeks SJW was not inferior to sertraline regarding HAMD. In follow-up phase, the HAMD score at the end of the study was 5.7 +/- 4.8 points for SJW and 7.1 +/- 6.3 points for sertraline. Comparable improvement was also found for the von Zerssen's Adjective Mood Scale (BfS) and CGI  The adverse events of 12 patients in the hypericum group (9.8 %) and of 16 patients in the sertraline group (13.6 %) were possibly related to study medication. No basic differences in the treatment groups were observed and no interaction with concomitant medication was documented. |
| Davidson et al. | 2002  JAMA | USA | 4 | Randomized, double-blind, controlled trial | 340 / 8 weeks | major depression / HAMD, CGI, | SJW extract (LI-160)  SJW 900 to 1500 mg/d or sertraline 50 - 100 mg/d or placebo | Placebo  Sertraline | HAMD was not different from placebo neither for sertraline nor H perforatum  Sertraline was better than placebo on the CGI improvement scale  Adverse-effect profiles for H perforatum and sertraline differed relative to placebo |
| Brenner et al. | 2000  Clinical Therapeutics | USA | 4 | double-blind, randomized pilot study | 30 / 1 plus 6 weeks | Mild to moderate depression / HAMD, CGI | SJW extract (LI 160)  SJW 600 mg/d or 50 mg/d sertraline for 1 week, followed by SJW 900 mg/d or sertraline 75 mg/d for 6 weeks. | Sertraline | HAMD and CGI were significantly reduced in both treatment groups. Clinical response (defined as a > or =50% reduction in HAM-D scores) was noted in 47% of patients receiving hypericum and 40% of those receiving sertraline. The difference was not statistically significant.  Both agents were well tolerated. |
| Gastpar et al. | 2005  Pharmaco-psychiatry | Germany | 4 | double-blind, randomized, multi-center clinical study | 241 / 12 weeks (+ 12 weeks) | Moderate depression / HAMD, BfS, CGI | SJW extract  (STW3)  SJW 612 mg or sertraline 50 mg/d | Sertraline | First 12-weeks: HAMD score decreased from almost identical initial values of about 22 points to about 8 points. SJW extract is not inferior to sertraline.  Second 12 weeks (Follow-Up), HAMD score at the end of the study was 5.7 +/- 4.8 points for SJW and 7.1 +/- 6.3 points for sertraline  Comparable improvement also for BfS and CGI in both treatment groups.  Tolerability of SJW extract and sertraline were "good" or "very good." |
| Rapaport et al. | 2011 / Journal of psychiatric research | USA | 4 | Randomized, blinded, controlled clinical trial | 169 screened 100 eligible 81 randomized/ 12 weeks (completed 59) | Minor depression / IDS-C, IDS-SR, HAMD, CGI-S, and Q-LES-Q total scores, 8 MOS scales, and 6 WBS scales | SJW 810 mg/d or citalopram 20 mg/d, or placebo | Placebo  Citalopram | No statistically significant differences between the groups concerning depressive symptom severity, quality of life, or well-being.  high placebo response on all outcome measures, 🡪 neither St. John’s Wort nor citalopram separated from placebo on change in depressive symptom severity, quality of life, or well-being.  No changes in MinD  SJW and citalopram were both associated with AEs during treatment |
| Gastpar et al. | 2006  Pharmaco-psychiatry | Germany | 4 | randomized, controlled, double-blind trial | 388 / 6 weeks | Moderate depression / HAMD, BfS, CGI | SJW extract (STW3-VI)  SJW 900 mg/d or citalopram 20 mg/d or placebo | Placebo  Citalopram | HAMD score was reduced (about 50%) for SJW and citalopram likewise and to less extend for placebo  🡪 statistical significant therapeutic equivalence of SJW to citalopram and the superiority of SJW over placebo  end of treatment 54.2 % (SJW), 55.9 % (citalopram) and 39.2 % (placebo) of the patients were assessed as therapy responders.  For BfS, CGI no differences between SJW and citalopram, but both were significantly superior to the placebo.  Significantly more adverse events were documented in the citalopram group |
| Singer et al. | 2011  Phytomedicine | Germany | 4 | randomized, multicentric, double-blind, placebo-controlled study | 154 responders / 6 weeks | moderate depression / HAMD | SJW extract (STW 3-VI)  SJW 900 mg/d or citalopram 20 mg/d or placebo | Citalopram  Placebo | numbers of patients with relapses were highest in the citalopram group (14 of 54), whereas patients who were treated with SJW showed the lowest relapse rate (8/54); patients from the placebo group showed a relapse rate of 8/46.  The duration of response was longest for the SJW group (1817 days), intermediate for the citalopram group (1755 days) and shortest for the placebo group (802 days). |
| Seifritz et al. | 2016  International Journal of Psychiatry in Clinical Practice | Switzerland | 4 | subgroup analysis of a double blind, randomized trial, | 64 patients / 6 weeks | Mild – moderate depression / HAM-D | SJW extract (WS 5570)  SJW 900 mg/d or paroxetine 20 mg/d | Paroxetine | Reductions of HAM-D total score were significantly more pronounced in patients treated with 900 mg/d SJW compared to 20 mg/d paroxetine.  Additionally, patients treated with SJW yielded greater response and remission rates compared with patients treated with paroxetine. |
| Anghelescu et al. | 2006 /  Pharmaco-psychiatry | Germany | 4 | double-blind, double-dummy, randomized continuation phase | 133 responders / 16 week | episode of moderate to severe depression / relapse prevention / HAMD, MADRS, BDI | SJW extract (WS 5570)  SJW 900 / 1800 mg / d or Paroxetine 20 / 40 mg/d | Paroxetine | HAMD total score decreased from 25.3+/-2.5 (mean+/-SD) to 4.3+/-6.2 points for SJW and from 25.3+/-2.6 to 5.2+/-5.5 points for paroxetine. Remission (HAMD endpoint total score below 8) occurred in 81.6% of the patients for SJW and in 71.4% for paroxetine.  Both SJW and paroxetine were well tolerated. |
| Szegedi et al. | 2005  BMJ-British Medical Journal | Germany | 4 | Randomised double blind, controlled, multicentre non-inferiority trial | 251 / 6 weeks | acute major depression / HAM-D, MADRS, CGI, BDI | SJW extract (WS 5570)  SJW 900 mg/d or paroxetine 20 mg/d | Paroxetine | HAMD decreased by 56.6% (SD 34.3%) of the baseline value, in the SJW group and by 44.8% (SD 33.5%) of baseline value in the paroxetine group. non-inferiority of hypericum and statistical superiority over paroxetine.  The incidence of adverse events was 0.035 and 0.060 events per day of exposure for SJW and paroxetine, respectively. |
| Fava et al. | 2005  Clin. Psycho-pharmacol. | USA | 4 | double-blind, randomized trial, controlled trial | 135 / 12 weeks | Major depression / HAMD, CGI, BDI | SJW extract (LI-160)  SJW 900 mg/d or fluoxetine 20 mg/d, or placebo | Fluoxetine  Placebo | lower mean HAMD-17 scores at end point in the SJW group compared with the fluoxetine group and a trend toward a similar finding relative to the placebo group  higher remission rates (HAMD-17 <8) in the SJW group (38%) compared with the fluoxetine group (30%) and the placebo group (21%).  Overall, SJW was safe and well tolerated. |
| Harrer et al. | 1999  Arzneimittel-forschung-Drug Research | Austria | 4 | randomised double-blind comparative trial | 149 elderly outpatients / 6 weeks | mild or moderate depressive episodes, HAMD | SJW extract (LoHyp-57)  SJW 800 mg/d or fluoxetine 20 mg/d | Fluoxetine | The efficacy of both medications was found to be equivalent both in mild and moderate depressive episodes.  Both treatment groups showed adverse drug reactions (ADRs): 12 ADRs for SJW and 17 ADRs for fluoxetine |
| Schrader | 2000  International Clinical Psycho-pharmacology | Germany | 4 | randomized, double-blind, parallel group comparison | 240 / 6 weeks | mild-moderate depression / HAMD, CGI, | SJW extract (Ze 117)  SJW 500 mg/d or fluoxetine 20 mg/d | Fluoxetine | Mean HAMD decreased to 11.54 on SJW and to 12.20 on fluoxetine, while mean CGI item I (severity) was significantly superior on SJW, as was the responder rate.  SJW safety was substantially superior to fluoxetine. |
| Friede et al. | 2001  Pharmaco-psychiatry | Germany | 4 | multicentric prospective randomized double-blind parallel group comparison | 240 / 6 weeks | mild to moderate depressive episodes / HAMD | SJW extract (Ze 117)  SJW 500 mg/d or fluoxetine 20 mg/d | Fluoxetine | HAMD responder rate was 60% for SJW compared to 40% for fluoxetine.  marked decrease of depressive agitation (pre-post comparison: 46%), depressive obstruction (44%), sleep disorders (43%) and anxiety symptoms (44%) during the therapy with SJW.  Adverse events occurred in 25% of the patients in the fluoxetine group and in 14% of the SJW group. |
| Bjerkenstedt et al. | 2005  Eur Arch Psychiatry Clin Neurosci | Sweden | 4 | randomized, placebo-controlled multi-center study | 160 / 4 weeks | Mild to moderate depression / HAMD, MADRS, CGI, Remission | SJW extract (LI 160)  900 mg SJW or 20 mg fluoxetine, or placebo / d | Placebo  Fluoxetine | No significant differences could be observed regarding efficacy measures except for remission rate (SJW 24%; fluoxetine 28%; placebo 7 %). Hypericum was significantly better tolerated than fluoxetine. SJW extract or fluoxetine were not more effective in short-term treatment in mild to moderate depression than placebo. |
| Moreno et al. | 2006  Braz J Psychiatry | Brazil | 4 | randomized double-blind trial | 72 / 8 weeks | Mild to moderate depression / HAMD, MADRS; CGI, Remission | Hypericum perforatum 900 mg, fluoxetine 20 mg or placebo | Fluoxetine  Placebo | No differences between the mean scores of the three groups. Patients receiving Hypericum perforatum had the lowest remission rates (12%) compared to fluoxetine (34.6%) and placebo (45%). |
| Vorbach et al. | 1994  J Geriatr Psychiatry Neurol | Germany | 4 | Multi-center, randomized, double blind comparative study | 135 / 6 weeks | Depression / HAMD, D-S, CGI | SJW extract (LI 160)  SJW 3 x 300 mg or 3 x 25 mg imipramine daily | Imipramine | In both treatment groups, a reduction of HAMD from 20.2 to 8.8 (SJW) or from 19.4 to 10.7 (imipramine), and the transformed D-S point values from 39.6 to 27.2 (SJW) and 39.0 to 29.2 (imipramine) were found. The analysis of CGI revealed comparable results in both treatment groups.  In the SJW group fewer and milder side effects were found as compared to imipramine. |
| Vorbach et al. | 1997  Pharmaco-psychiatry | Germany | 4 | randomized, controlled, multicentre double blind study | 209 / 6 weeks | severely depressed patients / HAMD, D-S, CGI | SJW extract (LI 160)  SJW 1800 mg/d or imipramine 150 mg/d | Imipramine | both treatment regimens very similar effective at the end of the 6 week-treatment period (mean values 25.3 to 14.5 in the SJW group and 26.1 to 13.6 in the imipramine group) regarding HAMD and CGI  Less adverse events were observed in the SJW group |
| Philipp et al. | 1999  British Medical Journal | Germany | 4 | Randomised, double blind, multicentre, parallel group trial | 263 / 8 weeks | moderate depression / HAMD, Zung self rating, CGI, SF-36 | SJW extract (STEI 300)  SJW 1050 mg/d or imipramine 100 mg/d  or placebo | Imipramin  Placebo | SJW was more effective at reducing HAMD than placebo and as effective as imipramine. Comparable results were found for Hamilton anxiety and CGI and were most pronounced for the Zung self rating depression scale. Quality of life (SF-36) was more improved with both active treatments than with placebo but in the physical component scale was improved only by hypericum extract compared with placebo.  The rate of adverse events with SJW was in the range of the placebo group but lower than that of the imipramine group |
| Woelk | 2000  BMJ | Germany | 4 | Randomised, multicentre, double blind, parallel group trial | 324 outpatients/ 6 weeks | mild to moderate depression / HAMD, CGI | SJW extract (ZE117)  SJW 500 mg/d or imipramine 150 mg/d | Imipramin | HAMD decreased in SJW group from 22.4 at baseline to 12.00 at end point and in the imipramine group from 22.1 to 12.75. Mean CGI at end point was 2.22 out of 7 for the SJW group and 2.42 for the imipramine group. None of the differences between treatment groups were significant. However, the mean score on the anxiety-somatisation subscale of the HAMD indicated a significant advantage for SJW relative to imipramine.  Tolerability was better for SJW than for imipramine. |
| Harrer et al. | 1994  J Geriatr Psychiatry Neurol | Austria | 4 | randomized, double-blind, multi-center study | 102 / 4 weeks | Depression / HAMD, D-S, CGI | SJW extract (LI 160)  3 x 300 mg of the SJW extract or 3 x 25 mg maprotiline | Maprotiline | HAMD dropped in both treatment groups by about 50%. The mean values of the D-S scale and the CGI scale showed similar results, and after 4 weeks of therapy, no significant differences in either treatment group were noticed. The onset of the effects occurred up to the second week of treatment, but were observed earlier with maprotiline than with SJW.  more cases of tiredness, mouth dryness, and heart complaints were observed for maprotiline |
| Wheatley | 1997  Pharmaco-psychiatry | United Kingdom | 4 | controlled, double blinded randomized, multicentre trial | 165 / 6 weeks | mild to moderate depression / HAMD, MADRS, CGI | SJW extract (LI 160)  SJW 900 mg/d or amitriptyline 25 mg/d | Amitriptyline | Depressive symptomatology was reduced substantially in both groups, and also with placebo; no statistically significant difference between the groups, although a tendency for a better response rate was seen in the amitriptyline group. HAMD and MADRS showed a significant advantage for amitriptyline, but only at week 6. Tolerability of SJW was clearly superior to amitriptyline, particularly in relation to anticholinergic and Central Nervous System adverse events. |
| Rhodiola rosea | | | | | | | | | |
| Mao et al. | Phytomedicine 2015 | USA | 4 | Phase II randomized, double-blind, placebo-controlled clinical trial | 57 / 12 weeks | mild to moderate major depressive disorder / HAMD, BDI, CGI/C | R. rosea extract (SHR-5)  R. rosea extract 340 mg or sertraline 50 mg or placebo | Placebo  Sertraline | Modest, statistically non-significant, reductions of HAMD, BDI, and CGI/C scores for all treatment conditions with no significant difference between groups. The decline in HAMD scores was greater for sertraline versus R. rosea and placebo. While the odds of improving (versus placebo) were greater for sertraline than R. rosea  More subjects on sertraline reported adverse events than R. rosea or placebo. |
| Darbinyan et al. | 2007  Nord J Psychiatry | Armenia | 4 | double-blind, randomized, parallel-group study | 89 / 6 weeks | Mild to moderate depression / HAMD | R. rosea extract (SHR-5)  R. rosea 340 mg/d or R. rosea 680 mg/d or placebo | Placebo | For patients in the R. rosea groups, overall depression, together with insomnia, emotional instability and somatization improved significantly following medication, whilst the placebo group did not show such improvements.  No serious side-effects were reported in any of the groups |
| **Sleeping Disorder / Insomnia** | | | | | | | | | |
| Valerian | | | | | | | | | |
| Donath et al., | 2000  Pharmaco-psychiatry | Germany | 4 | randomised, double-blind, placebo-controlled, cross-over study. | 16 / 14 days | psycho-physiological insomnia / percentage of time in bed (TIB) | Valerian extract (LI 156)  valerian 600 mg/d or placebo | Placebo | Sleep efficiency showed a significant increase for both the placebo and the valerian extract in comparison with baseline polysomnography.  In comparison with the placebo, slow-wave sleep latency was reduced after administration of valerian. The SWS percentage of time in bed (TIB) was increased after long-term valerian treatment, in comparison to baseline.  There was an extremely low number of adverse events during the valerian treatment periods. |
| Koetter et al. | 2007  Phytother Res | Switzerland | 4 | Randomized, Double Blind, Placebo-Controlled, Prospective Clinical Study | 30 /4 weeks | non-organic sleep disorder | single valerian extract (Ze 911) or a valerian hops extract combination (Ze 91019)  Valerian 500 mg/d or valerian & hops 500 mg & 120 mg/d or placebo | Placebo | The fixed extract combination was significantly superior to the placebo in reducing the sleep latency whilst the single valerian extract failed to be superior to the placebo. |
| Ziegler et al. | 2002  Eur J Med Res | Germany | 4 | randomized, double-blind, comparative clinical study | 202 / 6 weeks | non-organic insomnia / sleep quality (SF-B) | valerian extract LI 156  valerian 600 mg/d  oxazepam 10 mg/d | Oxazepam | Both treatments markedly increased sleep quality. SF-B subscales, i.e. feeling of refreshment after sleep, psychic stability in the evening, psychic exhaustion in the evening, psychosomatic symptoms in the sleep phase, dream recall, and duration of sleep confirmed similar effects of both treatments. CGI and Global Assessment of Efficacy by investigator and patient, showed similar effects of both treatments.  No serious adverse drug reactions were reported in either group. |
| M. Dorn | 2000  Forschende Komplementär-medizin Und Klassische Naturheilkunde 2000 | Germany | 4 | Randomised, double blind, comparative study | 75 / 28 days | Non-organic and non-psychiatric insomniacs / sleep quality SF-B | Valerian extract (LI 156)  valerian 600 mg/d  oxazepam 10 mg/d | Oxazepam | In both groups sleep quality improved but no statistically significant difference could be found between groups.  No serious adverse events happened. |
|  |  |  |  |  |  |  |  |  |  |
| Anxiety | | | | | | | | | |
| Lavendula | | | | | | | | | |
| Kasper et al. | 2015  Eur Neuropsycho-pharmacol | Austria / Germany | 4 | randomized, controlled double-blind trial | 170/ 10 weeks | anxiety-related restlessness and disturbed sleep / HAMA | Lavendula angustifolia extract (WS 1265)  Lavendula 80mg/d or placebo | placebo | HAMA total score decreased in the lavendula group and in all outcome measures the treatment effect of lavendula was more pronounced than with placebo.  Reporting of adverse events was equal in both groups. |
| Seifritz et al. | 2019  J Psychiatr Res | Switzerland | 4 | randomized, controlled double-blind trial | 212 / 10 weeks | Anxiety related disturbed sleep / HAMA, PSQI | Lavendula angustifolia extract (WS 1265)  Lavenula 80 mg/d or placebo. | placebo | Compared to placebo, lavendula significantly reduced the total scores of the HAMA and PSQI after ten weeks, with clinically meaningful differences that were observed already after two and six weeks for HAMA and PSQI, respectively. Lavendula had a statistically meaningful indirect effect on sleep (mediated by the effect on anxiety) but no appreciable direct effect. |
| Kasper et al. | 2010  Int Clin Psycho-pharmacol | Germany / Austria | 4 | randomized,controlled double-blind trial | 221 / 10 weeks | subsyndromal anxiety disorder / HAMA | Lavendula angustifolia (WS 1265)  Lavendula 80 mg/d or placebo | placebo | Patients treated with lavendula showed a decrease for the HAMA which was superior to placebo regarding the percentage of responders and remitters. Lavandula oil preparation had a significant beneficial influence on quality and duration of sleep and improved general mental and physical health without causing any unwanted sedative or other drug specific effects. |
| Kasper et al. | 2014  International Journal of Neuropsycho-pharmacology | Germany / Austria | 4 | randomized,controlled double-blind trial | 539 / 10 weeks | Generalized anxiety disorder / HAMA | Lavendula angustifolia (WS 1265)  Lavendula 160 or 80 mg/d paroxetine 20 mg/d or placebo | Paroxetine  placebo | HAMA decreased for both lavendula groups and the paroxetine group. Both lavendula groups were superior to placebo in reducing the HAMA total score  The difference between paroxetine and placebo was more pronounced regarding HAMA total score reduction.  lavendula extract showed a pronounced antidepressant effect and improved general mental health and health-related quality of life.  AE rates for lavendula were comparable to placebo and lower than for the active control paroxetine. |
| Kasper et al. | 2016  Eur Neuropsycho-pharmacol | Germany / Austria | 4 | randomized,controlled double-blind trial | 318 / 70 days | Mixed anxiety and depressive disorder / HAMA | Lavendula angustifolia (WS 1265)  Lavendula 80mg/d or placebo | placebo | Compared to placebo, the patients treated with lavendula extract had a better over-all clinical outcome and showed more pronounced improvements of impaired daily living skills and health related quality of life. Eructation was the only adverse event with a substantially higher incidence for lavendula extract. |
| Woelk & Schlafke | 2010  Phytomedicine | Germany | 4 | randomized,controlled double-blind trial | 77 /.6 weeks | Generalized anxiety / HAMA | Lavendula angustifolia (WS 1265)  Lavendula 80 mg/d  Lorazepam 0.5 mg/d | Lorazepam | HAMA decreased clearly and to a similar extent in both groups. During the active treatment period, the two HAMA subscores "somatic anxiety" (HAM-A subscore I) and "psychic anxiety" (HAM-A subscore II) also decreased clearly and to a similar extent in both groups. The changes in other subscores, such as the SAS (Self-rating Anxiety Scale), PSWQ-PW (Penn State Worry Questionnaire), SF 36 Health survey Questionnaire and CGI of severity of disorder, and the results of the sleep diary demonstrated comparable positive effects of the two compounds. |
| **Cognitive impairment & Alzheimer** | | | | | | | | | |
| Ginkgo bilboa | | | | | | | | | |
| Gavrilova et al | 2014  International Journal of Geriatric Psychiatry | Russia / Ukraine / Germany | 4 | multi-center randomized,controlled double-blind trial | 160 / 24 weeks | mild cognitive impairment with neuropsychiatric symptoms / NPI composite score | Ginkgo extract EGb 761  Ginkgo  240 mg / d or placebo | Placebo | NPI composite score decreased by 7.0 ± 4.5 points in the Ginkgo group and by 5.5 ± 5.2 in the placebo group. Improvement by at least 4 points was found in 78.8% of patients treated with Ginkgo and in 55.7% of those receiving placebo. Superiority of Ginkgo over placebo was also found for the State-Trait Anxiety Inventory score, the informants' global impression of change, and both Trail-Making Test scores. There were statistical trends favouring Ginkgo the Geriatric Depression Scale and the patients' global impression of change. Adverse events (all non-serious) were reported by 37 patients taking Ginkgo and 36 patients receiving placebo. |
| Gschwind et al. | 2017  Aging Clinical and Experimental Research | Switzerland / Germany | 4 | randomised, double-blind, placebo-controlled exploratory study | 50 / 6 months | mild cognitive impairment and associated gait impairment /  gait analyses | Ginkgo extract (LI 1370)  Ginkgo  240 mg/d or placebo | Placebo | 120 mg of Ginkgo twice-daily for at least 6 months may improve dual-task-related gait performance in patients with MCI. |
| Le Bars et al. | 2002  Neuro-psychobiology | USA | 4 | randomized, double-blind, placebo-controlled, parallel-group, multicenter study | 236 / 52 weeks | Cognitive impairment (diff. severities) or Alzheimer  ADAS-Cog, GERRI, CGI-C | Ginkgo extract EGb 761  Ginkgo 40 mg/d or placebo | Placebo | In the severity stratum 1 (MMSE >23), the placebo group did not show significant changes, while the Ginkgo group improved significantly by 1.7 points on the ADAS-Cog and by 0.09 points on the GERRI. In the severity stratum 2 (MMSE <24), the placebo group worsened by 4.1 points on the ADAS-Cog and 0.18 points on the GERRI, whereas the Ginkgo group showed 60% less decline on the ADAS-Cog and no change on the GERRI  The most severely impaired subgroup (MMSE <15) showed slightly more pronounced worsening for both treatment groups. However, in comparison to placebo, Ginkgo induced virtually the same magnitude of effect as was observed in the entire stratum 2.  Improvement was observed in the group of patients with very mild to mild cognitive impairment, while in more severe dementia, the mean Ginkgo effect should be considered more in terms of stabilization or slowing down of worsening, as compared to the greater deterioration observed with placebo. |
| Napryeyenko et al. | 2007  WMW | Ukraine | 4 | randomised, placebo-controlled, double-blind clinical trial | 400 / 22 weeks | Alzheimer dementia associated with neuropsychatric features  SKT test battery (9-item cognitive test battery) | Ginkgo biloba extract (EGb 761)  Ginkgo 240 mg/d or placebo | Placebo | There was a mean -3.2-point improvement in the SKT upon Ginkgo treatment and an average deterioration by +1.3 points on placebo. Ginkgo was significantly superior to placebo on all secondary outcome measures.  The drug was well tolerated; adverse events were no more frequent under drug than under placebo treatment. |
| Schneider et al. | 2005  Curr Alzheimer Res | USA | 4 | Randomized, placebo-controlled, double-blind, parallel-group, multicenter trial. | 513 outpatients / 26 weeks | mild to moderate dementia of the Alzheimer type / ADAS-cog, ADCS-CGIC | Ginkgo biloba extract (EGb 761)  Ginkgo 120 mg or 240 mg /d | Placebo | No significant between-group differences for the whole sample. There was little cognitive and functional decline of the placebo-treated patients, however. For a subgroup of patients with neuropsychiatric symptoms there was a greater decline of placebo-treated patients and significantly better cognitive performance and global assessment scores for the patients on Ginkgo |
| van Dongen et al. | 2003  Journal of Clinical Epidemiology | Netherlands | 4 | randomized, double-blind, placebo-controlled, parallel-group, multicenter trial | 214 / 12 weeks plus 12 weeks crossover in ginkgo groups | elderly patients with Alzheimer disease or vascular dementia or age-associated memory impairment  SKT test battery | Ginkgo biloba extract (EGb 761)  Ginkgo 240 mg/d or 160 mg/d) or placebo | Placebo | There were no statistically significant differences in mean change of scores between Ginkgo and placebo.  There was no dose-effect relationship and no effect of prolonged Ginkgo treatment. The trial results do not support the view that Ginkgo is beneficial for patients with dementia or age-associated memory impairment. |
| Le Bars et al. | 1997  JAMA | USA | 4 | randomized double-blind, placebo-controlled, parallel-group, multicenter study. | 309 included / 202 evaluable data / 52 weeks | Alzheimer disease and multi-infarct dementia.  ADAS cog score | Ginkgo biloba extract (EGb 761)  Ginkgo 120 mg /d or placebo | Placebo | The Ginkgo group had an ADAS-Cog score 1.4 points better than the placebo group and a GERRI score 0.14 points better than the placebo group. The same patterns were observed with the evaluable data set in which 27% of patients treated with Ginkgo achieved at least a 4-point improvement on the ADAS-Cog, compared with 14% taking placebo; on the GERRI, 37% were considered improved with Ginkgo, compared with 23% taking placebo. No difference was seen in the CGIC.  Regarding the safety profile of Ginkgo no significant differences compared with placebo were observed |
| Le Bars | 2003  Pharmaco-psychiatry | USA | 4 | Re-analysis of a randomized, double-blind, placebo-controlled study | 168 / 52 weeks | Alzheimer's disease stratified according to 23 MMSE cut offs (neuropsychological profiles)  ADAS cog score | Ginkgo biloba extract (EGb 761)  Ginkgo 120 mg/d or placebo | Placebo | At the endpoint, the Ginkgo group showed an improvement of 1.7 points according to ADAS-Cog and 0.15 points according to GERRI, while the placebo group worsened by 1.3 and 0.02 points, respectively. Retrospective analysis of overall efficacy indicated that a quantitative treatment effect favourable to Ginkgo could be observed in cognitive performance and social functioning, even taking NP differences and baseline severity into account. However, qualitative Ginkgo effects could greatly depend on NP profiles. Improvement could be expected with RAD patients, while Ginkgo effect should be considered more in terms of stabilization for GAD and delayed worsening for LAD population. |
| Nasab, et al. | 2012  Journal of the Pakistan Medical Association | Iran | 4 | randomized double blind study | 56 / 24 weeks | Alzheimer demenzia / Seven Minute test, Mini-Mental State Examination | Ginkgo biloba extract (EGb 761)  Ginkgo 120 mg/d or rivastigmine 4.5 mg /d | Rivastigmine | This study contributes to establish the efficacy and tolerability of the Ginkgo biloba in dementia of the Alzheimer type not as good as rivastigmine. Considering the evidence, it is suggested that cholinesterase inhibitors should be used in preference to Ginkgo biloba in patients with mild to moderate AD. |
| Mazza et al. | 2006  Eur J Neurol | Italy | 4 | randomized placebo-controlled double-blind study | 76 / 24 weeks | mild to moderate Alzheimer's dementia  MMSE, SKT text battery | Ginkgo biloba extract (EGb 761)  Ginkgo 160 mg /d or donepezil 5 mg /d or placebo | Placebo  Donepezil (Second generation cholinesterase inhibitor) | The results confirm the clinical efficacy of Ginkgo biloba in the dementia of the Alzheimer type, comparable with donepezil clinical efficacy. The study contributes to establish the efficacy and tolerability of the Ginkgo biloba in the dementia of the Alzheimer type with special respect to moderately severe stages. |

*Note.* The study scores of the quality assessment represent the following study type: 1 point for an observational study or a pre-post observational comparison, 2 points for a clinical trial, 3 points for a randomized controlled trial, 4 points for a blinded RCT

Results highlighted in green indicate positive effects of the herbal medicine, results marked in yellow indicate that the effects of the herbal medicine have not been superior to the comparison group and results marked in red indicate that the effects of the herbal medicine have been inferior to the comparison group

SJW = St. John’s wort, BDI = Beck Depression Inventory; D-S = Zerssen's Depression Scale, CGI = Clinical Global Impression; HAMD = Hamilton Depression Scale, GPA = global patient's self-assessment, MADRS = Montgomery-Asberg Depression Rating Scale, CSL-58 = Symptom Check List – 58, VAS = Visual Analogue Scale, GPA = Global Patients’ Self-Assessment, BfS = von Zerssen’s Adjective Mood Scale, IDS-C, Inventory of Depressive Symptomatology – Clinician-Reated, IDS-SR = Inventory of Depressive Symptomatology – Self-Report, Q-LES-Q = Quality of Life Enjoyment and Satisfaction Questionnaire, MOS, Mean Opinion Score, WBS = Well-Being Scale, ADR = Adverse Drug Reaction, PSQI = Pittsburgh Sleep Quality Index, GAD = Generalized Anxiety Disorder, MCI = Mild Cognitiv Impairment, GERRI = Geriatric Evaluation by Relative’s Rating Instrument, MMSE = Mini-Mental State Examination, ADCS-CGIC = Alzheimer's Disease Cooperative Study-Clinical Global Impression Of Change, Rad = Right Alzheimer’s Disease, LAD = Left Alzheimer’s Disease, GAD = General Alzheimer’s Disease, SKT = German: *Syndrom-Kurztest*, Cognitive Test Battery, ADAS-Cog = Alzheimer's Disease Assessment Scale-Cognitive Subscale
