# Supplementary material for: Current state of research on the clinical benefits of herbal medicines for non-life-threatening ailments
Source: Front Pharmacol. 2023 Sep 28;14:1234701. doi: 10.3389/fphar.2023.1234701 (PMC10569491; doi:10.3389/fphar.2023.1234701)
Supplement: Supplementary file 2 [file Table2.docx]

| **Gynecological complaints** | | | | | | | | | |
| --- | --- | --- | --- | --- | --- | --- | --- | --- | --- |
| Authors/  reference | Year of publication/ Journal | Country | Study score^a^ | Study design | Population (N)/ Duration | Indication/ Outcome | Treatment | Comparison | Results^b^ |
| **Menopausal symptoms** | | | | | | | | | |
| Black cohosh | | | | | | | | | |
| Bai et al. | 2007  Maturitas | China | 4 | randomized, double-blind, parallel-controlled study | 244 / 3 months | Menopausal symptoms  Kupperman Menopause Index (severity of menopausal symptoms)  Benefit-risk-balance | Isopropanolic black cohosh extract  2.5 mg  ≙ 20 mg herbal drug | Tibolone  2.5 mg | The benefit-risk value (ratio of KMI score/number of adverse events) revealed significant non-inferiority (p<.001) and superiority (p=.014) of black cohosh. |
| Briese et al. | 2007  Maturitas | Germany | 1 | prospective, controlled open-label observational study | 6141 / at least one dose | Menopausal symptoms  Menopause Rating Scale subscore PSYCHE (severity of psychological symptoms) | Isopropanolic black cohosh extract  2.5 mg  ≙ 20 mg herbal drug | Combination therapy of 3.75 mg isopropanolic black cohosh extract and 70 mg St. John’s wort extract | Significant lower Menopause Rating Scale subscore PSYCHE in both groups with significant higher reduction for the combination therapy.  Rate of treatment-related adverse events was 0.23% for the monotherapy group and 0.10% for the combination therapy group. |
| Drewe et al. | 2013  Phytomedicine | Switzerland | 1 | observational study | 442 / 3 months high dose + 6 months continuation or low dose (decided by physician) | Menopausal complaints  Kupperman Menopause Index (severity of menopausal symptoms) | High dose black cohosh extract  Ze 450  13 mg | Low dose black cohosh extract  Ze 450  6.5 mg | Kupperman Menopause Index decreased significantly in 3-month treatment phase with higher dose.  Adverse events were experienced by n=16 in the high dose group and by n=2 in the low dose group. |
| Friederichsen et al. | 2020  Archives of gynecology and obstetrics | Switzerland | 1 | retrospective observational study | 174 / Mean follow up: 12 months | Menopausal symptoms  Metabolic serum parameters, body weight,  Menopausal Rating Scale (severity of menopausal symptoms) | Black cohosh extract  Ze 450  6.5 mg or 13 mg | Menopausal hormone therapy | Menopausal Rating scale vegetative and urogenital scores decreased significantly in both groups. |
| Juliá Mollá et al. | 2009  Gynecological endocrinology | Spain | 1 | prospective observational study | 122 / 3 months | Post-menopausal amenorrhea, hot flushes  Cervantes Hr-QoL scales (menopause-specific quality of life) | Isopropanolic black cohosh extract  2.5 mg  ≙ 20 mg herbal drug | No comparison | Significant increase in quality of life according to Cervantes scale scores (except for couple relationship domain. |
| Liske et al. | 2002  Journal of women's health & gender-based medicine | Germany, Switzerland | 4 | controlled, randomized, double-blinded parallel group study | 152 / 24 weeks | Peri- and postmeno-pausal women  Kupperman Menopause Index (severity of menopausal symptoms) | High dose  isopropanolic black cohosh extract  127.3 mg herbal drug | Standard dose isopropanolic black cohosh extract  39 mg herbal drug | Kupperman Menopause Index decreased significantly independent of dose.  Rates of adverse events were equal in both groups. |
| Nappi et al. | 2005  Gynecological endocrinology | Italy | 3 | randomized clinical study, | 64 / 3 months | Menopausal complaints  Hot flushes per day, Greene scale (burden of menopausal symptoms), Symptom Rating Test (anxiety and depression) | Isopropanolic black cohosh extract  40 mg | Transdermal estradiol 25 μg + 10 mg dihydrogesterone for the last 12 days | Significant (p<.001) reduction of hot flushes per day, vasomotor symptoms, and anxiety and depression with no significant difference between both treatment groups. |
| Osmers et al. | 2005  Obstetrics and gynecology | Germany | 4 | randomized, multicenter, double-blind  clinical trial | 304 / 12 weeks | Menopausal complaints  Menopausal Rating Scale (severity of menopausal symptoms) | Isopropanolic black cohosh extract  2.5 mg  ≙ 20 mg herbal drug | Placebo | Significant higher decrease in Menopause Rating Scale score for the treatment with black cohosh (p=.027).  Number of patients with adverse events was not significant between groups (p=.771). |
| Vermes et al. | 2005  Advances in therapy | Hungary | 1 | pre-post observational study | 2016 / 12 weeks | Menopausal symptoms with refusal or contra-indication for hormone replacement therapy  Kupperman Menopause Index (severity of menopausal symptoms) | Isopropanolic black cohosh extract  2.5 mg | No comparison | Clinically relevant decrease in Kupperman Menopause Index score of 17.64 points in average (p<.001).  Rate of unexpected adverse events was 12.1%. |
| Wuttke et al. | 2003  Maturitas | Germany | 4 | double-blind, randomized, controlled multicenter study | 62 / 3 months | Menopausal symptoms  Menopausal Rating Scale (severity of menopausal symptoms) | Black cohosh extract  BNO 1055  40 mg herbal drug | Conjugated estrogens  0.6 mg  Placebo | Decrease in Menopause Rating Scale score between black cohosh and conjugated estrogens was not significant. Difference to placebo just approached significance (p=.051).  Number of participants with adverse events was similar in the three groups. No serious adverse events occurred. |
| Sage | | | | | | | | | |
| Bommer et al. | 2011  Advances in therapy | Switzerland, Germany | 2 | open clinical trial | 72 / 1 baseline week, 8 weeks treatment | Menopausal hot flushes  Intensity and frequency of hot flushes, mean number of intensity-rated hot flushes | 280 mg holistic, thujone-free sage spissum extract, equivalent 3400 mg tincture of fresh sage leaves | No comparison | The proportion of mild hot flushes increased, whereas proportions of moderate, severe and very severe hot flushes was reduced. Decrease of mean number of hot flushes was significant (p<.001).  Treatment-related adverse events occurred in only one participant and were mild. |
| **Pre-menstrual syndrome** | | | | | | | | | |
| Vitex agnus castus | | | | | | | | | |
| Berger et al. | 2000  Archives of gynecology and obstetrics | Switzerland, Germany | 1 | prospective, open-label study | 43 / 2 cycles baseline, 3 cycles treatment, 3 cycles without treatment | PMS  Moos’ menstrual distress questionnaire (disability due to premenstrual symptoms) | Vitex agnus castus extract  Ze 440  20 mg | No comparison | Significant reduction in Moos’ menstrual distress questionnaire (p<.001). Symptoms returned after finishing the treatment, but were still reduced compared to baseline.  Adverse events occurred in 20 participants. |
| He et al. | 2009  Maturitas | China | 4 | prospective, double-blind, placebo controlled, parallel-group, multi-center clinical trial | 217 / up to 3 cycles | PMS  Premenstrual syndrome diary (severity of premenstrual symptoms) | Vitex agnus castus extract  BNO 1095  4 mg  ≙ 40 mg herbal drug | Placebo | Mean PMSD score difference at the end of treatment was significant between groups in the favor of VAC.  The difference between adverse events rates was not significant. |
| Lauritzen et al. | 1997  Phytomedicine | Germany | 4 | multicenter, controlled, randomized, comparative  trial | 175 / 3 cycles | PMTS (pre-menstrual tension syndrome)  PMTS scale (severity of premenstrual symptoms) | Vitex agnus castus extract  BNO 1095  3.5-4.2 mg | Pyridoxine  100 mg | Decrease in PMTS score was higher for the VAC group than for the pyridoxine group.  Adverse events occurred in n=5 for the VAC group and in n=4 for the pyridoxine group. |
| Loch et al. | 2000  Journal of women's health & gender-based medicine | Germany | 1 | multicentric noninterventional trial (open study without control) | 1634 / 3 cycles | PMS  Frequency of investigator-documented PMS symptoms | 1.6-3.0 mg dried Vitex agnus castus extract | No comparison | Frequency of all documented complaints decreased significantly (p<.001). 41.6% of participants had no PMS symptoms at all at the end of the treatment.  Adverse events occurred in 18 participants. |
| Ma et al. | 2010  The Australian & New Zealand journal of obstetrics & gynaecology | China | 4 | prospective, randomized, double-blind, placebo-controlled study | 67 / 3 cycles | PMS  Premenstrual Syndrome Diary (severity of premenstrual symptoms) | Vitex agnus castus extract  BNO 1095  4 mg  ≙ 40 mg herbal drug | Placebo | Differences in PMSD scores between groups were significant for all symptoms (p<.001).  An adverse event occurred in only one person (treatment group). |
| Momoeda et al. | 2014  Advances in therapy | Japan | 1 | multi-center, prospective, open-  label, single-arm, phase 3 study | 69 / 3 cycles | PMS  VAS total score (intensity of premenstrual symptoms) | Vitex agnus castus extract  Ze 440  20 mg | No comparison | Mean VAS total score decreased significantly from cycle to cycle (p<.001).  Adverse events occurred in eight participants of which none was serious. |
| Schellenberg^18^ | 2001  BMJ | Germany | 4 | randomized, double blind, placebo controlled, parallel group comparison | 178 / 3 cycles | PMS  VAS total score (intensity of premenstrual symptoms) | Vitex agnus castus extract  Ze 440  20 mg | Placebo | VAS total score decreased in both groups. The difference between groups was significant (p=.001).  In the treatment group, n=4 adverse events occurred and n=3 in the placebo group. |
| Schellenberg et al.^19^ | 2012  Phytomedicine | Germany, Switzerland | 4 | multicenter, double-blind, placebo-controlled, parallel-group study | 162 / 3 cycles | PMS  VAS total score (intensity of premenstrual symptoms) | Vitex agnus castus extract  Ze 440  20 mg | Placebo  Vitex agnus castus extract  8 mg Ze 440  30 mg Ze 440 | Symptom improvement was significantly higher for the reference dose (20 mg) compared to placebo or the lose dose (8 mg); no significant difference between treatment with 20 mg or 30 mg  The number of participants with adverse events was as follows: placebo: n=3; 8 mg: n=2; 20 mg: n=1; 30 mg: n=4. |
| St. John’s wort | | | | | | | | | |
| Canning et al. | 2010  CNS Drugs | UK | 4 | randomized, double-blind, placebo-controlled, crossover  study | 36 / 2 cycles | mild PMS  Daily Symptom Report Score (severity of premenstrual symptoms) | Hypericum perforatum extract  Li 160  450 mg | Placebo | Significant improvement of physical and behavioral PMS symptoms treated with St. John’s wort compared to placebo (p=.04).  20 adverse events were reported for the placebo group and 15 for the treatment group. |

*Note.* The study scores of the quality assessment represent the following study types: 1 point for an observational study or a pre-post observational comparison; 2 points for a clinical trial; 3 points for an RCT; 4 points for a blinded RCT

Results highlighted in green indicate positive effects of the herbal medicine and results marked in yellow indicate that the effects of the herbal medicine have not been superior to the comparison group.

Hr-QoL: Health-related Quality of Life; KMI: Kupperman Menopause Index; PMS: Premenstrual syndrome; PMSD: Premenstrual symptom diary; PMTS: Premenstrual tension syndrome; VAC: Vitex agnus castus; VAS: Visual Analogue Scale.
