# Supplementary material for: Current state of research on the clinical benefits of herbal medicines for non-life-threatening ailments
Source: Front Pharmacol. 2023 Sep 28;14:1234701. doi: 10.3389/fphar.2023.1234701 (PMC10569491; doi:10.3389/fphar.2023.1234701)
Supplement: Supplementary file 3 [file Table5.docx]

| Upper respiratory infections | | | | | | | | | |
| --- | --- | --- | --- | --- | --- | --- | --- | --- | --- |
| Authors / reference | Year of publi-cation | Country | Study score | Study design | Popula-tion (N)/ Duration | Indication / Outcome | Treatment | Comparison | Results |
| **Bronchitis** | | | | | | | | | |
| Chuchalin AG,et al | 2005  Explore (NY) | Russia | 4 | Randomized, double blind, controlled trial | 124 / 7 days | **Acute bronchitis / Bronchitis** Severity Score | Pelargonium sidoides (EPs 7630)  3x30 drops/day (4.5 mL per day) | placebo | The decrease of BSS from baseline to day seven was 7.2 ± 3.1 points with EPs 7630 and 4.9 ± 2.7 points with placebo. 95% CI for the difference of effects between the two treatment groups was 1.21- 3.56, showing a significant improvement of EPs 7630 compared with placebo on day seven.  A very good or good tolerability was reported by 98.4% of the patients in the EPs 7630 group and by 96.7% of the patients in the placebo group. A total of 25 of 124 patients (20.2%) experienced at least one AE during the trial: 15 of 64 patients (23.4%) in the EPs 7630 group and 10 of 60 patients (16.7%) in the placebo group, with intensities ranging from mild to moderate. |
| Gillissen A, et al | 2013  Drug Res (Stuttg) | Germany | 4 | Double-blind, randomized, placebo-controlled, multicenter trial | 398 / 2 weeks | **Acute bronchitis**, Frequency of day-time coughing fits and BSS | Eucalyptus, sweet orange, myrtle and lemon oil; ELOM-080, 4x300 mg/day (1200 per day) | placebo | There was a significantly change in coughing fits of 62.1 % (95 % CI: 57.6–66.6 %) under ELOM-080 treatment compared to 49.8 % (95 % CI: 44.6–55.0 %) under placebo. The mean changes in BSS from baseline were larger at each on-treatment visit in the patients treated with ELOM-080 than in the patients treated with Placebo; at all visits, the treatment difference was statistically significant. |
| Hecker M,et al | 2002  Forsch Komplementarmed Klass Naturheilkd | Germany | 1 | multicenter, prospective post-marketing surveillance (PMS) study | 1350 / 4 weeks | **Chronic bronchitis** / clinical symptoms and tolerability | Hederae folium extract; EA-575  Dose 2x65 mg/day (130 mg per day) | NA | In particular symptoms of illness-related productive cough showed a relevant improvement in over 90% of study participants during the 4 weeks of observation, with just over 40% reporting complete remission of symptoms.  Adverse events were reported by 0,4% of the patients. This was once nausea (resulting in study stop) and twice reflux a relation to study medication could not be ruled out. |
| Kähler C, et al | 2019  Wien Med Wochenschr | Austria + Poland | 4 | Randomized, double blind, controlled trial | 229 / 10 days | **uncomplicated acute bronchitis** **/** BSS | Pelargonium sidoides, EPs 7630. 2x3 capsules/day (900 mg per day) | placebo | The mean decrease in BSS at day 7 and day 10 was significant with 4.79 vs. 3.20 and 6.47 vs. 4.32 score points respectively in the intervention vs. placebo group . |
| Matthys H, Heger M. | 2007  Phytomedicine | Germany? | 1 | multicenter, prospective, open outcomes  study | 205 / 7 days | **acute bronchitis or acute exacerbation of chronic bronchitis** / Symptom score (SS), max 20 points. | Pelargonium sidoides, EPs 7630 80g/100 ml, 3x30 drops/day (90 drops per day) | NA | SS amounted to 6.1±2.8 points on average at the start of treatment and decreased to 2.8±2.6 points by the final examination on day 7.  18 AE in 7.8% of the patients were documented, eleven involving the gastrointestinal tract. Of these, three were classified as suspected adverse drug reactions, although a relationship with the test medication was assessed as ‘‘unlikely’’. Serious adverse events did not occur. |
| Matthys H, Heger M. | 2007  Curr Med Res Opin | Russia | 4 | randomised, double-blind,  placebo-controlled, multicentre  study | 217 / 7 days | **acute**  **bronchitis** /change in bronchitis  symptom score (BSS) | Pelargonium sidoides 80g/100 ml, EPs 7630, 3x30 drops/day (90 drops per day) | placebo | After 7 days of treatment, the BSS decreased by 7.6 ± 2.2 points in the EPs 7630 group and by 5.3 ± 3.2 points in the placebo group. There was a highly significant superiority for the EPs 7630 treatment.  No serious AEs were recorded. A total of 47/217 patients (21.7%) experienced at least one AE during the trial, 23/108 patients (21.3%) in the active treatment group and 24/109 patients (22.0%) in the placebo group. Most frequently coded were ‘investigations’ and ‘blood and lymphatic disorders’, including laboratory abnormalities, such as increase in the erythrocyte sedimentation rate (EPs 7630, 10/108 patients [9.3%]; placebo, 10/109 patients [9.2%]) and changes in leucocyte count (EPs 7630, 4/108 patients [3.7%]; placebo, 5/109 patients [4.6%]) were particularly frequent, which are due to the underlying infectious disease. The number of AEs did not differ substantially between the two treatment groups. |
| Matthys H,et al | 2010  Curr Med Res Opin | Ukraine | 4 | a randomised, double-blind, placebo-controlled dose-finding study with 4 arms | 406 / 7 days | **acute**  **bronchitis** /change in bronchitis  symptom score (BSS) | Pelargonium sidoides, EPs 7630: 3x10mg/day or 3x20mg/day or 3x30mg/day (30 mg/day or 60 mg/day or 90 mg/day) | placebo | Between day 0 and day 7, the mean BSS score decreased by 2.7 ± 2.3 (placebo), 4.3 ± 1.9 (30-mg group), 6.1 ± 2.1 (60-mg group), and 6.3 ± 2.0 points (90-mg group), respectively. The differences between the EPs 7630 groups and placebo were statistically significant.  92 mild or moderate AEs were observed in 18.5% of patients, mainly affected was the System Organ Class ‘gastrointestinal disorders’ (6/102 (5.9%) patients in the placebo group, 5/102 (4.9%) in the 30-mg group, 9/101 (8.9%) in the 60-mg group and 15/101 (14.9%) in the 90-mg group). None of the AEs was classified as serious. |
| Matthys H,et al | 2003  Phytomedicine | ? | 4 | Randomized, double blind, controlled trial | 468 / 7 days | **acute**  **bronchitis** /change in bronchitis  symptom score (BSS) | Pelargonium sidoides 80g/100 ml, EPs 7630: 3x30 drops/day (90 drops per day) | placebo | The decrease of BSS from baseline to day 7 was 5.9 ± 2.9 points under EPs 7630 (n = 233), and 3.2 ± 4.1 points under placebo (n = 235). There was a significant superiority of EPs 7630 compared to placebo on day 7.  7,7% of the patients experienced at least one adverse event (AE) during the trial, 8.6 % of the patients in the EPs 7630 group and 6.8 % of the patients in the placebo group. All adverse events were assessed as non-serious. In the EPs 7630-treated group 15 patients reported 22 mild adverse events and in the placebo group 9 mild adverse events were reported by 8 patients. |
| **Common cold, Acute Sinusitis / acute Rhinosinusitis** | | | | | | | | | |
| Barrett BP et al | 2002  Ann Intern Med | USA | 4 | Randomized, double blind, controlled trial | 148 / 10 days | **Common cold** 7 Severity and duration of self-reported symptoms of upper respiratory tract infection. | *E. angustifolia* root (123 mg), *E. purpurea* root (62 mg), *.E. purpurea* herb (62 mg),  Dose 4x6 capsules on day1, then 3x6 day (2214 mg resp. 2952 mg E. *angustifolia,* 1116 mg resp. 1488 mg *E. purpurea* root*,* and *1116 mg resp 1488 mg E. purpurea* herb per day*)* | Placebo | No statistically significant differences between the intervention and placebo groups for any of the measured outcomes.  Specific adverse effects were reported 22 times by 15 participants who completed the study (9 times by 7 participants in the placebo group and 13 times by 8 participants in the intervention group). In the intervention group, sleeplessness, heartburn, nausea, stomachache, and upset stomach were each reported by one participant and bad taste was noted by three participants. In the placebo group, stomachache was noted by three participants and nausea, belching, thirst, and abdominal pain with diarrhea were each noted by one participant. Reported adverse effects were therefore not statistically different between the intervention and placebo groups. |
| Barrett B,et al | 2010  Ann Intern Med | USA | 4 | RCT with four parallel groups: 1) no pills, 2) placebo pills (blinded), 3) echinacea pills (blinded), or 4) echinacea pills (open-label). | 719 (713 completed) / 14 days | **Common cold** / global severity | Tablets contained the equivalent of 675 mg *E. purpurea* root standardized to 2.1mg alkamides and 600 mg *E.angustifolia* root standardized to 2.1mg alkamides. Dose 4x2 capsules on day1, then 4x1 day (5400 mg resp. 2700 mg *E. purpurea* root and 4800 mg resp. 2400 mg *E.angustifolia* root per day) | placebo | Severity and illness duration were lower in the blinded and open-label intervention groups than in either the blinded placebo or no pill groups, but no significant differences.  Frequency of potential adverse effects was similar (statistically indistinguishable) in the four groups. The only possible exception was headache, where 62% of those in the no pill group reported having had a headache at some time during their illness, compared to less than 50% in the three pill groups. |
| Naser B et al | 2005  Phytomedicine | Italy | 4 | randomized, double-blind, placebo-controlled, clinical dose–response trial | 91 / 3–12 days | **common cold** / total number of facial tissues used throughout the clinical duration of their cold | Baptisia tinctoria root/ Echinacea purpurea root/Echinacea pallida root and Thuja; DER 4–9:1.  Dose: 13x9,2mg/day or 3x9,6mg /day (119,6 mg or 28,8 mg per day) | placebo | The total number of facial tissues used throughout the observational period decreased with increasing dose of extract administered. The slope across groups according to the Jonckheere test was significant for the primary efficacy parameter in both the ITT and PP analyses. Time to relevant improvement in cold symptoms (measured as the time until less than 30 tissues per day were used) was 1.1 days, 0.76 days and 0.52 days in the placebo, low-dose and high-dose groups, respectively.  No adverse events were reported. |
| Yale SH, Liu K | 2004  Arch Intern Med | USA | 4 | Randomized, double blind, controlled trial | 128 / 14 days | **Common Cold** / total symptom scores (SS) | *E. Echinacea* from the aerial portion of E purpurea and standardized for a content of 2.4% soluble β-1,2-D-fructofuranosides. Dose 3x1 capsules/day (300 mg per day) | placebo | No statistically significant difference was observed between treatment groups for total SS.  A few adverse events were reported, with headache and dry mouth being the predominant adverse effects in both treatment groups, although this may not accurately reflect adverse events because headache is also a frequent symptom of the common cold. |
| Goel et al | 2004  Journal of Clinical Pharmacy and Therapeutics | Canada | 4 | Randomized, double blind, controlled trial | 128 / 7 days | **Common cold**, Total Daily Symptom Scores (TDSS): summing the daily scores for 13 symptoms. Primary end point: change in TDSS for the 7-day period. | The extracts were combined in 40% ethanol to provide  an echinacea formulation containing alkamides/ cichoric acid/polysaccharides at concentrations of 0.25/2.5/25.5 mg/mL, respectively. Dose: 10 equally distributed throughout day 1, then 4 doses/day (10 mg, 100 mg or 1000 mg at day 1; 4 mg, 40 mg or 400 mg per day from day 2) | placebo | The self-assessed TDSS were significantly lower in the intervention group. In the ITT population, the effect of the intervention on primary efficacy parameter resulted in 17.6% reduction in TDSS. In both populations, ITT and PP, overall mean severity scores (mean of 7 days) for all specific symptoms, except for cough, were found to be lower in the intervention group.  The treatments were well tolerated by the subjects. Gastrointestinal (nausea, heartburn and constipation) side effects were reported by 13% of those taking study medication and 9% taking placebo. Other effects such as itching, burning sensation and numbness of the tongue, were reported by 13% and 11% for study medication and placebo recipients, respectively. However, none of the subjects withdrew from the trial because of these side effects. Evidence for development of secondary complications such as bronchitis was found in two in the intervention and five in the placebo groups. |
| Bachert C et al | 2009  Rhinology | Ukraine | 4 | Randomized, double blind, controlled trial multicenter trial with a group-sequential adaptvie design | 103 / 7 days | Change in **Sinusitis** Severity Score (SSS) after 7 days | Pelargonium sidoides, EPs 7630, dose 3x3ml per day (9 ml per day) | placebo | Mean decrease in SSS: 5.5 point in the Eps 7630 group compared to 2.5 point in the placebo group, a change difference of 3.0 points (95% confidence interval 2.0 to 3.9.  No clinically relevant changes in any laboratory safety parameter. A total of 7.8% patients reported at least one adverse event (AE) during the trial, 11.8% in the EPs 7630 group and 3.8% patients in the placebo group. All AEs were assessed as non-serious. |
| Dejaco D et al | 2019  Rhinology | Austria + Poland | 4 | Randomized, double blind, controlled trial multicenter trial | 288 / 8 days | **Viral acute Rhisosinusitis (ARS**) / Major Symptom Score (MSS) | Pelargonium sidoides, EPs 7630, dose Capsule 150 mg Spicae aetheroleum.  Dose: 3x2 per day (900 mg per day) | placebo | After 7 days of full medication, the patients in the Eps 7630 group had a significantly lower MSS compared to placebo .  A total of 39 adverse events (AEs) were reported by 34 patients during the study. In the Eps 7630 arm 26/147 patients and in the control arm 8/141 had an AE. Two out of the 30 AEs from the Eps 7630 group have been rated as probably and 20 as possibly related; Of the 30 AEs from the Eps 7630 group, 19 were reported as mild compared to 7/9 in the placebo. Twenty-one patients in the Eps 7630 group reported 23 gastrointestinal disorders including nausea (4), abdominal pain (14), appendicitis (1), upper abdominal pain (1), diarrhoea (1), breath odour (1) and dysgeusia (1). One case of abdominal pain and the one with appendicitis were both assessed as unrelated, all others with exception of upper abdominal pain and dysgeusia (probably related) were rated as possibly related to study medication. No new safety signals were identified, no adverse event was classified as serious. |
| Perić A et al | 2020  Ann Otol Rhinol Laryngol | Serbia | 3 | Prospective, randomized, open-label, non-inferiority study | 50 / 10 days | **Uncomplicated Acute Bacterial Rhinosinusitis** / total symptom score (TSS) | Pelargonium sidoides, EPs 7630, 3x 20 mg/day (60 mg per day) | Amoxicillin, 3x 500 mg/day | Higher absolute improvement after treatment was found for TSS in EPs 7630 group compared to amoxicillin group.  None of the patients in both groups reported any adverse events. |
| Federspil P et al | 1997  Laryngorhinootologie | Germany | 4 | Randomized, double blind, controlled trial multicenter 3-arm trial | 331 / 2 weeks | **Acute sinusitis** / adapted Sinusitis Severity Score | Eucalyptus, sweet orange, myrtle and lemon oil; ELOM-080 (300 mg) or another essential oil (300 mg) plus Xylometazolin dosing spray. Dose: 4x 300 mg ELOM-080 or 4x 300 mg other essential oil + 4x2 puffs Xylometazolin Dosing spray | placebo | The intervention groups showed a statistically significant decrease in the symptom score as compared to placebo for both interventions: There was a medication stop in 10 of 33 persons in the group ‚other ‘essential oils‘, which was found in only 3 cases of the ELOM-080-group and 2 in the placebo group. 28 adverse events in the expected spectrum, could be timely linked to study medication, were observed. |
| Gottschlich S et al | 2018  Advances in therapy | Germany | 2 | Prospective, Non-interventional Parallel-Group Trial | 228 / 2 weeks | **Acute Rhinosinusitus (ARS)** / facial pain relief | Eucalyptus, sweet orange, myrtle and lemon oil; ELOM-080. Dose: 3-4 capsules per day (900mg – 1200 mg per day) | Gentianae radix, Primulae flos, Sambuci flos, Rumicis herba and verbenae herba, BNO 1016. Dose: 3x2 tablets per day (72 Gentianae radix mg, 216 mg Primulae flos, 216 mg Sambuci flos, 216 mg Rumicis herba, 216 mg verbenae herba per day) | Pain relief in patients treated with ELOM-080 was more than 1 day (1.2 days) ahead when compared to the BNO 016 group. The mean score after 2 weeks of treatment was calculated as 0.47 for ELOM-080 and was statistically significant lower (p = 0.0147) compared to the mean score of 0.93 for BNO 1016.  During treatment, 12 ADRs were documented for 7 patients during treatment with ELOM-080 and 4 ADRs were documented for 4 subjects during treatment with BNO 1016. In total 15 of the 16 ADRs were classified as possibly treatment related. Of these 11 ADRs were assessed to be possibly related to the treatment with ELOM-080, and 4 ADRs were possibly related to the treatment with BNO 1016. ADRs possibly related to ELOM-080 mainly comprised mild gastrointestinal disorders (see below) and repeated dizziness or headache in two patients Intensity of the ADRs was mild (12 ADRs) or not reported (4 ADRs). No ADR was assessed to be serious. |
| Jund R, et al | 2015  Acta Otolaryngol | Belgium? | 3 | randomized placebo-controlled clinical trial | 589 / 14 days | **Acute viral Rhinosinusitis (ARS)** / Major Symptom Score (MSS) | Gentianae radix, Primulae flos, Sambuci flos, Rumicis herba and verbenae herba; BNO 1016, dose 3x160 mg daily (480 mg per day) | Placebo | MSS improved during the treatment period by a mean of 10.02 ± 1.61 score points to 2.47 ± 2.55 for BNO 1016 and of9.87 ± 1.52 to 3.63 ± 3.63 for placebo. Differences between treatment groups at end of therapy were statistically significant in favor of BNO 1016.  Serious adverse events (SAEs) were not reported. In ARhiSi-1 a total of 42 AEs occurred in 33 patients of the SEP (safety evaluable population): 33 AEs in 8.7% of the patients under treatment with BNO 1016 (2 treatment groups with BNO 1016 in a daily dose of 240 mg or 480 mg) and 9 AEs in 4,7% patients under treatment with placebo. In ARhiSi-2 a total of 53 AEs occurred in 46 patients of the SEP: 21 AEs in 9.8% patients under treatment with BNO 1016 480 mg and 32 AEs in 14.1% patients under treatment with placebo. The majority of AEs reported under BNO 1016 were mild to moderate |
| Jund R et al | 2015  MMW Fortschr Med | Germany? | 4 | Randomized, double blind, controlled trial multicenter trial | 386 / 14 days | **Acute viral Rhinosinusitis (ARS)** / Major Symptom Score (MSS) | Gentianae radix, Primulae flos, Sambuci flos, Rumicis herba and verbenae herba; BNO 1016, dose 3x160 mg daily (380 mg per day) | placebo | Treatment with BNO 1016 was associated with a statistically significant and clinically relevant difference in favor of the herbal drugs group.  During the study 9.8% of patients in the intervention group experienced an adverse event, as did 14.1% of patients receiving placebo. All adverse events were mild. |
| Passali D et al | 2015  ORL J Otorhinolaryngol Relat Spec | ? | 1 | multicenter, prospective, open-label study | 60 / 14 days | **Acute Rhinosinusitis (ARS)** / major symptom scores according to investigator (MSS inv ) and the Health-Related Quality of Life score. | Gentianae radix, Primulae flos, Sambuci flos, Rumicis herba and verbenae herba; BNO 1016, 3x 1 tablet daily (108 mg Sambuci flos + 108 mg Primulae flos + 108 mg Rimicis herba + 108 mg Verbenae herba + 36 mg Gentianae radix per day) | intranasal fluticasone furoate, 2 puffs in each nostril, once a day | All patients in the study showed significant improvements in their symptom score from the beginning of treatment, and this improvement continued and increased for the entire duration of the treatment with both drugs. The difference in the results is not statistically significant.  None of the patients of the intervention group reported any adverse events, and all their vital signs were normal. Among the patients of the control group, 1 patient had epistaxis, and 2 patients reported nasal itching. However, all patients of this group had normal vital signs. |
| **Chronic rhinosinusitis** | | | | | | | | | |
| Palm J, et al | 2017  Rhinology | Belgium, Czech  Republic, Germany and Poland | 4 | multicentre, Randomized, double blind, controlled trial parallel group trial | 929 / 12 weeks | **chronic rhinosinusitis** / Major Symptom Score (MSS) in week 8 and week 12 | BNO 1016, 240 mg extract/day or 480 mg extract/day | placebo | BNO 1016 extract was not superior over placebo regarding the primary endpoint.  In total, 1,215 AEs were reported by 57 % patients during the treatment period and 150 adverse events by 11.7% during the follow-up. Most of the events were mild to moderate. A relationship with the study drug was suspected in 4.4% of the cases treated with BNO 1016 240 mg; in 5.9% patients treated with the 480 mg and in 3.9 % patients who received placebo.  Serious adverse events were noted in 1.2% patients during the treatment period and in 0.5% patients during the follow- up period. No causal relationship with the study medication was seen for all of these. |
| **Other URIs** | | | | | | | | | |
| Martin D, et al | 2020  Postgrad Med | Germany | 1 | retrospective cohort study / IMS® Disease Analyzer database | 234.364  Results reported on 169.528 adult patients | **Acute lower and upper tract respiratory infections** / subsequent prescription of antibiotics + duration of sick leave | Prescribed phytopharmaceuticals | Without phytopharmaceuticals | Extract EPs 7630 and thyme extract compared to no phytopharmaceutical prescription exhibited the strongest decrease in antibiotics prescriptions among patients treated by general practitioners (non-pediatric patients).  Phytopharmaceutical prescription on the day of diagnosis was significantly associated with fewer long sick leaves The strongest associations were found for cineole and EPs 7630 |
| Schaefer A, et al | 2016  Pharmazie | Germany | 4 | Randomized, double blind, controlled trial multi-center trial | 181 / 1 week | **acute cough** /cough severity (CS) assessed by Visual Analogue Scale (VAS) | Ivy leaves dry extract; EA 575.  Dose 3x5 ml per day (105 mg per day) | placebo | Significant difference between the two study groups with regard to the primary outcome the mean AUC0-168 h was 7902.6 mm*h in the verum group and 9637.3 mm*h in the placebo group. Higher values of the AUC0-168 h indicate a higher severity of cough.  AEs occurred in 11.6 % subjects (active treatment: n=9, placebo: n=12). 18 subjects had one single AE, one subject in the active treatment group and one subject in the placebo group had two AEs each, another subject in the placebo group had four AEs. All reported AEs were relatively well-balanced between the treatment groups and are closely connected to the underlying disease as cough (“worsening of cough”), middle ear effusion, and sinusitis. It was mainly at one of the five sites where the investigator evaluated slight worsening of 2 to 5 mm on VAS in the cough assessment as AEs. However, in most of these cases BSS and VCD remained at the same level. |
|  |  |  |  |  |  |  |  |  |  |

*Note.* The study scores of the quality assessment represent the following study types: 1 point for an observational study or a pre-post observational comparison; 2 points for a clinical trial; 3 points for an RCT; 4 points for a blinded RCT

Results highlighted in green indicate positive effects of the herbal medicine and results marked in yellow indicate that the effects of the herbal medicine have not been superior to the comparison group.

Abbreviations: SS = Symptom Score, BSS = Bronchitis Symptom Score, TDSS = Total Daily Symptom Score, SSS = Sinusitis Severity Score, ARS = Acute Rhinosinusitis, MSS = Major Symptom Score, TSS = Total Symptom Score, VAS = Visual Analogue Scale
