# Supplementary material for: Current state of research on the clinical benefits of herbal medicines for non-life-threatening ailments
Source: Front Pharmacol. 2023 Sep 28;14:1234701. doi: 10.3389/fphar.2023.1234701 (PMC10569491; doi:10.3389/fphar.2023.1234701)
Supplement: Supplementary file 4 [file Table3.docx]

| **Urinary Tract Infection (UTI) and Lower Urinary Tract Symptoms (LUTS)** | | | | | | | | | |
| --- | --- | --- | --- | --- | --- | --- | --- | --- | --- |
| Authors | Year of publication / Journal | Country | Study score | Study design | Population (N) / Duration | Indication / Outcome | Treatment | Comparison | Results |
| BNO 1045 | | | | | | | | | |
| Sabadash et al. | 2017  [Clinical Phytoscience](https://clinphytoscience.springeropen.com/) | Ukraine | 3 | Open labeled, randomized, controlled study | 90 / 3 months | Young women with acute recurrent cystitis caused by E. coli. Endpoints: Cystitis symptoms, pyuria, bacteriuria, LUTI Index | BNO 1045* 108 mg/drug/d plus antibacterial therapy for 7 days (Ofloxacin 400 mg/d) | Ofloxacin | Bacteriuria and recurrent episodes of cystitis were prevented more frequently in the test group, compared to the control group. |
| Kulchavenya | 2018 Therapeutic Advances in Urology | Russia | 1 | Pilot, open, noncomparative prospective study | 29 / 1 month | Young women with acute uncomplicated cystitis. Endpoints: Cystitis symptoms, dysuria, bacteriuria | BNO 1045 108 mg/drug/d plus Ketoprofen, 100 mg/d for 5 days | No comparison | 21 patients responded well to therapy with no dysuria and leucocyturia. 4 patients responded partially and 4 patients did not respond to therapy. |
| Miotla et al. | 2018  Biomed Research International | Poland | 1 | Observational study | 72 / 1 week | High-risk women with mixed urinary incontinence, neurogenic bladder, or unclear LUTS. Primary endpoints: UTI symptoms, pyuria, bacteriuria | BNO 1045 90 mg/d or Fosfomycin trometamol single shot | Fosfomycin trometamol | No statistical differences in the incidence of UTI between both treatment groups. 10 patients in the BNO 1045 group required continuing therapy for further 7 days. |
| Wagenlehner et al. | 2018  Urologia Internationalis | Germany | 4 | Double-blind, controlled, double-dummy, parallel-group, randomized, multicenter, multinational phase III non-inferior trial | 659 / 7 days | Women with acute uncomplicated cystitis. Primary endpoint: Additional use of antibiotics | BNO 1045 108 mg/drug/d and matched placebo, or Fosfomycin trometamol single shot and matched placebo | Fosfomycin trometamol | Non-inferiority of BNO 1045 over Fosfomycin trometamol in the treatment of acute uncomplicated UTI |
| Rechberger et al. | 2020  Journal of Clinical Medicine | Poland | **3** | Prospective, randomized single center study | 562 / 3 weeks | Women who underwent MSS due to stress urinary incontinence. Primary endpoint: percentage of clinically diagnosed and treated UTI. | BNO 1045 90 mg/d or Ciprofloxacin 1500 mg/d (for 3 days after surgery) | Ciprofloxacin | No statistically significant difference between the two groups concerning the number of patients developing UTI after MSS |
| Höller et al. | 2021  Antibiotics (Basel) | Germany | **1** | Retrospective database analysis | 160,912 / 31-365 days | Males and females with acute cystitis. Endpoints: UTI recurrence, UTI-related sick leave, and antibiotic prescriptions | BNO 1045^&^ | Antibiotic | Compared to antibiotics, BNO 1045 was significantly associated with fewer recurrences of UTI and with reduced additional antibiotic prescription. |
| Rechberger et al. | 2022  [European Journal of Obstetrics & Gynecology and Reproductive Biology](https://www.sciencedirect.com/journal/european-journal-of-obstetrics-and-gynecology-and-reproductive-biology) | Poland | 3 | Prospective, randomized single center study | 320 / 3 weeks | Women who underwent MSS due to stress urinary incontinence. Primary endpoint: Percentage of clinically diagnosed and treated UTI | BNO 1045 90 mg/d or combination of D-mannose + Arctostaphylos uva-ursi, Betula pendula and Berberis aristata 80 mg/d | BNO 1045 vs combination of D-mannose + Arctostaphylos uva-ursi, Betula pendula and Berberis aristata | Non-inferiority of combination of D-mannose + Arctostaphylos uva-ursi, Silver birch (Betula pendula) and Indian barberry (Berberis aristata) over BNO 1045 concerning the prevention of UTI after MSS. |
| Wawrysiuk et al. | 2022  Pathogens | Poland | 3 | Prospective, randomized, noninferiority experimental trial | 125 / 14 days | Women who underwent urogynecological surgeries. Primary endpoint: Positive urine analysis or UTI symptoms | BNO 1045 15 ml/d or Fosfomycin trometamol single shot | Fosfomycin trometamol | Non-inferiority of BNO 1045 over Fosfomycin trometamol concerning the prevention of UTI after surgery. |
| WS 1473/1031 and further standardized herbal extracts | | | | | | | | | |
| Albrecht et al. | 2007  Current Medical Research and Opinion | Germany | 4 | Prospective randomized, double-blind, placebo-controlled, multicenter trial | 219 / 90 days | Patients suffering from chronically recurrent UTI symptoms. Primary endpoint: Clinically confirmed recurrences | Armoraciae rusticanae 320 mg/d and Tropaeoli majoris 800 mg/d | Placebo | Recurrent UTI symptoms were less, compared to the placebo group. |
| Stange et al. | 2017  Research and Reports in Urology | Germany | 4 | Prospective, randomized, double-blind, double-dummy, multicenter, phase III clinical study | 96 / 7 days | Acute uncomplicated cystitis. Primary endpoint: Reduction of urinary pathogens | Armoraciae rusticanae 1600 mg/d and Tropaeoli majoris 4000 mg/d or Cotrimoxazol (320 mg/d trimethoprim and 1600 mg/d sulfamethoxazole)+ placebo | Cotrimoxazol | Responder rates, improvement of UTI symptoms, and cystitis relapse rates were similar in both groups. Non-inferiority could not be assumed due to slow recruitment rates. |
| Lopatkin et al. | 2005  [World Journal of Urology](https://link.springer.com/journal/345) | Russia | 4 | Prospective, double-blind, randomized, multicenter clinical trial | 257 / 48 weeks | Males suffering from LUTS caused by BPH. Primary endpoint: I-PSS score | WS 1473/1031** 320 mg/d 120 mg/d or placebo for 24 weeks, followed by a further 24 weeks WS 1473/1031 administration (all patients) | Placebo | WS 1473/1031 was superior to the placebo for the amelioration of LUTS. |
| Engelmann et al. | 2006  Arzneimittelforschung | Germany | 4 | Prospective, randomized, double-blind, double-dummy, multicenter trial | 140 / 60 weeks | LUTS caused by BPH. Primary endpoint: I-PSS score | WS 1473/1031  320 mg/d 240 mg/d or Tamsulosin | Tamsulosin | Non-inferiority of WS 1473/1031 over Tamsulosin in the treatment of LUTS caused by BPH. |
| Lopatkin et al. | 2007  International Urology and Nephrology | Russia | 4 | Prospective, randomized, double-blind, double-dummy, multicenter trial. Follow up of Lopatkin et al. 2005. | 257 / 96 weeks | LUTS caused by BPH. Primary endpoint: I-PSS score | WS 1473/1031  320 mg/d 240 mg/d for 48 weeks after completion study [Lopatkin et al. 2005] | No comparison | Treatment with WS 1473/1031 provided a clinically relevant benefit over a period of 96 weeks. |
| Oelke et al. | 2014  [World Journal of Urology](https://link.springer.com/journal/345) | Germany | **1** | Post hoc evaluation of four published randomized, double-blind clinical trials. | 922 / 24 weeks | LUTS caused by BPH. Primary endpoint: I-PSS score | WS 1473/1031  320 mg/d 240 mg/d or placebo or Finasteride or Tamsulosin | Finasteride, tamsulosin, placebo | WS 1473/1031 significantly improved nocturnal voiding frequency compared to placebo and was non-inferior to Tamsulosin or Finasteride. |
| Madersbacher et al. | 2022  Postgraduate Medicine | Germany | **1** | Retrospective cohort study | 77,923 / 12 months | Urinary incontinence, polyuria (including nocturia), urinary retention, and erectile dysfunction. Endpoint: Association between Prostagutt prescription and clinical response | WS 1473/1031 | Finasteride, Dutasteride, Tamsulosin, or Tamsulosin/Dutasteride combination | Significant association between WS 1473/1031 prescription and reduced incidence of urinary incontinence retention compared to tamsulosin and tamsulosin/dutasteride, and reduced incidence of erectile dysfunction compared to dutasteride. |
| Gauruder-Burmester et al. | 2019  Planta Medica | Germany | **2** | Noninterventional multicenter study | 113 / 12 weeks | Women with overactive bladder. Endpoints: Overactive bladder symptoms and ICIQ‑OAB score | Cucurbita pepo 681.9 mg/d Rhus aromatic 168 mg/d Humulus lupulus 54.0 mg/d | No comparison | Treatment progressively improved overactive bladder symptoms and related quality of life. |

*Note.* The study scores of the quality assessment represent the following study types: 1 point for an observational study or a pre-post observational comparison; 2 points for a clinical trial; 3 points for an RCT; 4 points for a blinded RCT

Results highlighted in green indicate positive effects of the herbal medicine and results marked in yellow indicate that the effects of the herbal medicine have not been superior to the comparison group.

Abbreviations: BPH: Benign prostatic hyperplasia. ICIQ: International consultation on incontinence modular questionnaire. IPSS: International prostate symptom score. LUTI: Lower urinary tract infection. LUTS: Lower urinary tract symptoms. MSS: Midurethral sling surgery. OAB: Overactive bladder. UDS: Urodynamic studies. UTI: Urinary tract infection.

* BNO 1045 contains 54 mg of a standardized herbal combination in a 1:1:1 mixture (BNO 2103) in one tablet: Centaurium erythraea Rafin s.l., herba; Levisticum officinale Koch, radix; Rosmarinus officinalis L., folium.

# 5 ml BNO 1045 contains 90 mg of a standardized herbal combination in a 1:1:1 mixture (BNO 2103).

& BNO 1045 double concentrated (108 mg in a 1:1:1 mixture).

** Each capsule contains 160 mg Sabal fruit extract (WS 1473) and 120 mg Urtica root extract (WS 1031).
