# Supplementary material for: Current state of research on the clinical benefits of herbal medicines for non-life-threatening ailments
Source: Front Pharmacol. 2023 Sep 28;14:1234701. doi: 10.3389/fphar.2023.1234701 (PMC10569491; doi:10.3389/fphar.2023.1234701)
Supplement: Supplementary file 5 [file Table4.docx]

| **Gastrointestinal diseases / dyspepsia** | | | | | | | | | |
| --- | --- | --- | --- | --- | --- | --- | --- | --- | --- |
| Authors | Year of publication / Journal | Country | Study score | Study design | Population (N) / Duration | Indication / Outcome | Treatment | Comparison | Results |
| **Hepatic diseases** | | | | | | | | | |
| Silymarin | | | | | | | | | |
| Adeyemo et al. | 2013  [Journal of Viral Hepatitis.](https://www.ncbi.nlm.nih.gov/pmc/articles/PMC3675799/) | USA | 4 | Multicenter, randomized, placebo-controlled, double-masked, prospective clinical trial | 32 / 20 weeks | Hepatitis C and non-alcoholic Steatohepatitis C / Serum ALT and HCV RNA | Silybum marianum (V03AB48) 1260 or 2100 mg/d | Placebo | Serum ALT and HCV RNA titers did not change in Silymarin or placebo group. |
| Fried et al. | 2012 Journal of the American Medical Association | USA | 4 | Multicenter, double-blind, placebo-controlled trial | 154 / 24 weeks | Hepatitis C / ALT levels; HCV RNA levels; QOL measures | Silybum marianum (V03AB48) 1260 or 2100 mg/d | Placebo | The mean decline in serum ALT activity did not differ significantly, there likewise were no significant differences in HCV RNA levels or QOL measures. The adverse event profile of silymarin was comparable with that of placebo. |
| Tanamly et al. | 2014  Digestive and Liver Disease | USA | 4 | Randomized double-blinded trial | 177 / 12 months | Hepatitis C / QOL; clinical trial using the short form-36 | Silybum marianum 373.5 mg/d | Placebo | Subjects reported feeling better, although symptoms and QOL scores did not differ between the silymarin and multivitamin groups. |
| Hawke et al. | 2009 Journal of Clinical Pharmacology | USA | 2 | Randomized 3:1, via Web-based randomization, were blinded to treatment assignments until trial completion | 32 / 7 days | Hepatitis C / safety and dose-exposure relationships of higher than customary oral doses of silymarin; acute effects on serum HCV RNA in noncirrhotic HCV patients | Silybum marianum 420, 840, 1680 or 2100 mg/d | Placebo | No drug-related adverse events were reported, and no clinically meaningful reductions from baseline serum transaminases or HCV RNA titer were observed compared to placebo. |
| Fathalah et al. | 2017  Journal of Interferon & Cytokine Research | Egypt | 4 | Randomized-controlled double-blind trial | 80 / 12 weeks | Hepatitis C / Serum aspartate aminotransferase, ALT; direct bilirubin; Serum albumin; Child scoring; QOL questionnaire | Silybum marianum 420 or 1050 mg/d | Dose comparison | High-dose regimen of silymarin had a positive impact on improving QOL. No serious adverse events were reported. |
| Parés et al. | 1998  Journal of Hepatology | Spain | 4 | Controlled, double-blind, randomized, and multicenter trial | 200 / 2 years | Cirrhosis of the liver / time to death | Silybum marianum 450 mg/d | Placebo | Silymarin did not have any significant effect on the course of the disease compared to placebo. No relevant side-effects were observed in any group. |
| Ferenci et al. | 2008  Gastroenterology | Austria | 1 | Observational study | 36 / 24 weeks | Chronic hepatitis C not responding to PEGylated interferon/ribavirin therapy / virologic response | Silybum marianum 10 mg/kg/d intravenously, change to Silybum marianum 420 mg/d  or Silybum marianum 5, 10, 15, 20 mg/kg/d intravenously, change to Silybum marianum 840 mg/d | Dose-finding study | Silibinin is well tolerated and shows a substantial antiviral effect against HCV in nonresponders. |
| Tanwar et al. | 2017  European Journal of Gastroenterology & Hepatology | UK | 1 | Randomized to receive with or without silymarin | 93 /  24 months | Chronic hepatitis C who had previously failed antiviral therapy / HCV RNA; Ishak fibrosis stage and ELF score | Silybum marianum 840 mg/d | PEGylated interferon with or without silymarin + placebo | The addition of silymarin did not influence outcome (HCV RNA; Ishak fibrosis stage and ELF score) |
| **Inflammatory Bowel Disease (IBD)** | | | | | | | | | |
| Potentilla erecta or Artemisia absinthium | | | | | | | | | |
| Huber et al. | 2007  Journal of Clinical Gastroenterology | Germany | 1 | Open-label, Dose-escalating study | 16 / 3 weeks | Ulcerative Colitis / side effects, CAI, C-reactive protein, and tannin levels in patient sera | Potentilla erecta  1200, 1800, 2400 or 3000 mg/d | No comparison | During therapy, the CAI decreased in all patients, whereas it increased during the washout phase.  Tormentil appeared safe up to 3000 mg/d. |
| Krebs et al. | 2010  Phytomedicine | Germany | 2 | Controlled trial with randomly selected patients | 20 / 6 weeks | Crohn’s disease / CDAI, IBDQ, and 21-item HAMD | Artemisia absinthium  2250 mg/d | Placebo | Wormwood showed reduction of tumor necrosis factor alpha, reduced CDAI scores, and improved IBDQ and HAMD, compared to the control. |
| Omer et al. | 2007  Phytomedicine | USA | 4 | Double-blind placebo-controlled | 40 / 10 weeks | Crohn’s disease / CDAI; IBDQ, HAMD; 8-item Visual Analogue Scale | Artemisia absinthium  1500 mg/d | Placebo | Wormwood has a steroid sparing effect. The improvements in HAMD scores indicate that wormwood also has an effect on the mood and QOL. |
| **Irritable Bowel Syndrome (IBS)** | | | | | | | | | |
| STW 5 + STW 5-II or Hypericum perforatum | | | | | | | | | |
| Madisch et al. | 2004 Alimentary Pharmacology and Therapeutics | Germany | 4 | Double-blind, randomized, placebo-controlled, multi-center trial | 208 / 4 weeks | IBS / changes in total abdominal pain and IBS symptom scores | STW 5 or STW 5-II 60 drops/d | Bitter candytuft mono-extract and placebo | STW 5 and STW 5-II were significantly better than placebo in reducing the total abdominal pain score and the IBS symptom score at 4 weeks. There were no statistically significant differences between the bitter candytuft mono-extract group and the placebo group. |
| **Saito et al.** | 2010  American Journal of Gastroenterology | USA | 4 | **Double-Blind, Randomized, Placebo-Controlled Trial** | **70 / 12 weeks** | **IBS /** self-reported BSS; and diarrhea-BSS, constipation-BSS, pain or discomfort, and bloating; adequate relief of IBS; and IBS QOL-score | Hypericum perforatum **900 mg/d** | Placebo | Both groups reported decreases in overall BSS from baseline, with the placebo arm having significantly lower scores at 12 weeks compared with Hypericum perforatum. |
| **Functional dyspepsia (FD)** | | | | | | | | | |
| WS1340 + WS1520, STW5 and/or STW 5-II | | | | | | | | | |
| Storr et al. | 2022  Zeitschrift für Gastroenterologie | Germany | 1 | Open-label follow-up of a randomized, double-blind, placebo-controlled study | 70 / 29 days | FD / absolute intra-individual changes in the patient ratings of pain intensity, PHF and CGI | WS 1340 180 mg/d or WS 1520 100 mg/d | Placebo | WS1520 + WS1340 treatment was well tolerated.  All efficacy variables were statistically significantly improved in the WS1520 + WS1340 group compared to placebo (PHF, global improvement). |
| Rich et al. | 2017 Journal of Neurogastroenterology and Motility | Germany | 3 | Randomized placebo-controlled | 114 / 4 weeks | Chronic or recurrent FD / health-related QOL using, NDI, Likert scales measuring frequency, intensity, and burdensomeness; pain intensity score; discomfort intensity score; EPS and PDS; VAS; CGI | WS 1340 180 mg/d or WS 1520 100 mg | Placebo | WS1520 + WS1340 is an effective therapy for the relief of pain and discomfort and improvement of disease-specific QOL in patients with functional dyspepsia and significantly improves symptoms consistent with EPS and PDS. |
| Madisch et al. | 1999  Arzneimittelforschung | Germany | 4 | Multicenter, reference-controlled double-blind equivalence study | 120 / 4 weeks | FD / VAS, PHF, bowel-associated symptoms, DDS; CGI | WS 1340 180 mg/d or WS 1520 100 mg/d reference preparation cisapride 30 mg/d | Cisapride and placebo | The combination WS1340/WS1520 appears to be comparable with cisapride and provides an effective means for treatment of functional dyspepsia. Both medications were tolerated well. |
| von Arnim et al. | 2007  American Journal of Gastroenterology | Germany | 4 | Multicenter, placebo-controlled, double-blind study | 315 / 8 weeks | FD / GIS | STW 5 60 drops/d | Placebo | STW 5 is effective and well tolerated in patients with FD. The study shows a significant superiority of STW 5 versus placebo in relieving dyspeptic symptoms after 4 and 8 weeks of treatment |
| Madisch et al. | 2004  Digestion | Germany | 4 | Double-blind, randomized, placebo-controlled, multicenter trial | 120 / 8 weeks | FD / GIS | STW 5-II 60 drops/d | Placebo with switch to active and vice versa | In patients with functional dyspepsia, the herbal preparation improved dyspeptic symptoms (GIS) significantly better than placebo. |
| Rösch et al. | 2002  Zeitschrift für Gastroenterologie | Germany | 4 | Double-blind, double-dummy study | 186 / 4 weeks | FD / GIS; efficacy and tolerability assessments, recurrences and safety parameters | STW 5 or STW 5-II 60 drops/d or cisapride 30 mg/d | Cisapride and placebo | STW 5 and STW 5-II showed equivalent efficacy to cisapride for the treatment of patients with FD of dysmotility type. |

Abbreviations: ALT: Alanine transaminase; BSS: Bowel Symptom Score ;CAI: Clinical Activity Index; CDAI: Crohn’s Disease Activity Index; CGI: Clinical Global Impressions; DDS: Dyspeptic Discomfort Score; ELF: Enhanced Liver Fibrosis; EPS: Epigastric Pain Syndrome; GIS: Gastrointestinal Symptom Score; HAMD: Hamilton's Depression Scale scores; HCV: hepatitis C virus; IBS: Irritable Bowel Syndrome; IBDQ: Inflammatory Bowel Disease Questionnaire Scores; NDI: Nepean Dyspepsia Index; PDS: Postprandial Distress Syndrome; PHF: Pressure, Heaviness and Fullness; QOL: quality-of-life; VAS: Visual Analog Scales
